# Reprocessable and Recyclable Materials for 3D Printing via Reversible Thia‐Michael Reactions

**DOI:** 10.1002/anie.202423522

**Published:** 2025-01-28

**Authors:** Yong‐Liang Su, Liang Yue, McKinley K. Paul, Joseph Kern, Kaitlyn S. Otte, Rampi Ramprasad, H. Jerry Qi, Will R. Gutekunst

**Affiliations:** ^1^ School of Chemistry and Biochemistry Georgia Institute of Technology Atlanta Georgia 30332 United States; ^2^ School of Mechanical Engineering Georgia Institute of Technology; ^3^ School of Materials Science and Engineering Georgia Institute of Technology

**Keywords:** Michael addition-elimination, ring-opening polymerization, chemically recyclable polymer, 3D printing, fused-filament fabrication

## Abstract

The development of chemically recyclable polymers for sustainable 3D printing is crucial to reducing plastic waste and advancing towards a circular polymer economy. Here, we introduce a new class of polythioenones (PCTE) synthesized via Michael addition‐elimination ring‐opening polymerization (MAEROP) of cyclic thioenone (CTE) monomers. The designed monomers are straightforward to synthesize, scalable and highly modular, and the resulting polymers display mechanical performance superior to commodity polyolefins such as polyethylene and polypropylene. The material was successfully employed in 3D printing using fused‐filament fabrication (FFF), showcasing excellent printability and mechanical recyclability. Notably, PCTE−Ph retains its tensile strength and thermal stability after multiple mechanical recycling cycles. Furthermore, PCTE−Ph can be depolymerized back to its original monomer with a 90 % yield, allowing for repolymerization and establishing a successful closed‐loop life cycle, making it a sustainable alternative for additive manufacturing applications.

## Graphical abstract

A 3D printing material platform based on polythioenones (PCTE) is introduced. The thioenone monomers are synthesized in a scalable two‐step process and are readily amenable to diversification. PCTE−Ph exhibits excellent tensile strength, machinability, and recyclability, achieving 90 % depolymerization yield in a closed‐loop polymerization/depolymerization cycle. Its performance in fused‐filament fabrication (FFF) highlights its potential for advanced additive manufacturing.

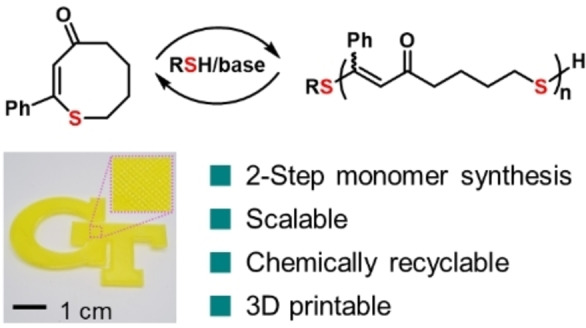

Synthetic polymers have become integral to modern life, providing essential materials for various applications. However, the ever‐growing consumption of plastics has led to significant environmental and economic challenges, particularly due to their persistent nature and the insufficient avenues available for recycling.[^1^, ^2^, ^3^, ^4^] In response to these concerns, the development of chemically recyclable polymers has emerged as a promising solution. These innovative materials can be depolymerized back into their original monomers when subjected to a suitable stimulus, thus establishing a circular “monomer‐polymer‐monomer” lifecycle.[^5^, ^6^, ^7^, ^8^, ^9^] This closed‐loop chemical cycle has the potential to revolutionize the lifecycle of plastics, mitigate the detrimental impact of plastic pollution, and reduce the reliance on fossil fuels for polymer production.

The integration of 3D printing technology with polymer science has expanded the horizons of material design and manufacturing. As an additive manufacturing technique, 3D printing enables the precise construction of complex structures layer by layer from digital models.[^10^, ^11^, ^12^] This capability allows for unparalleled customization and rapid prototyping, drawing significant attention in fields such as biomedical engineering, automotive manufacturing, and consumer product design.[^13^, ^14^] However, the intrinsic contradiction between the polymerization and depolymerization, along with the performance requirements of the materials, make it challenging to develop recyclable polymers suitable for 3D printing.[^15^, ^16^, ^17^] To this end, a strategy centered on monomer design is crucial for facilitating the efficient synthesis of high‐performance polymers while also enhancing their recyclability.

Ring‐opening polymerization (ROP) is a widely used chain‐growth polymerization technique that enables the preparation of polymers with well‐controlled structures.[^18^, ^19^, ^20^] Recently, ROP has been utilized in the development of recyclable polymers by targeting cyclic monomers with modest ring strain.[^21^, ^22^, ^23^] Several recyclable polymer platforms including polyesters,[^24^, ^25^, ^26^] polythioesters,[^27^, ^28^, ^29^, ^30^] polythioethers,[^31^, ^32^] polyacetals,[^33^, ^34^] polycarbonates,^[35]^ polyolefins,[^36^, ^37^] and others,[^38^, ^39^] have been developed through ROP, though very few have been explored in additive manufacturing.[^40^, ^41^] Despite these advances, there is a strong demand for new recyclable materials that offer performance comparable to, or even better than, commercial non‐recyclable polymers, while also maintaining suitability for 3D printing.[^42^, ^43^, ^44^, ^45^] The thia‐Michael addition is a type of click reaction that has been used in the synthesis of various polymers through step‐growth polymerization.[^46^, ^47^, ^48^, ^49^, ^50^, ^51^] Although few reports have explored its use in ROP, these systems typically lack recyclability or have limited recovery efficiency, hindering true circularity and their suitability for 3D printing remains unknown.[^32^, ^52^]

Herein, we report a new family of cyclic thioenone (CTE) monomers that are capable of ROP by leveraging the reversibility of the thia‐Michael addition reaction (Figure 1). The monomer scaffold can be easily synthesized from abundant alkyne sources and is readily modified. The ROP provides polythioenones (PCTE) that exhibit superior performance compared to common commercial plastics such as polypropylene (PP), low‐density polyethylene (LDPE) and high‐density polyethylene (HDPE). Moreover, the new plastic PCTE−Ph has been successfully utilized in 3D printing, can be reprocessed with negligible impact on its properties, and can be efficiently recycled back to the constituent monomers.

**Figure 1:**
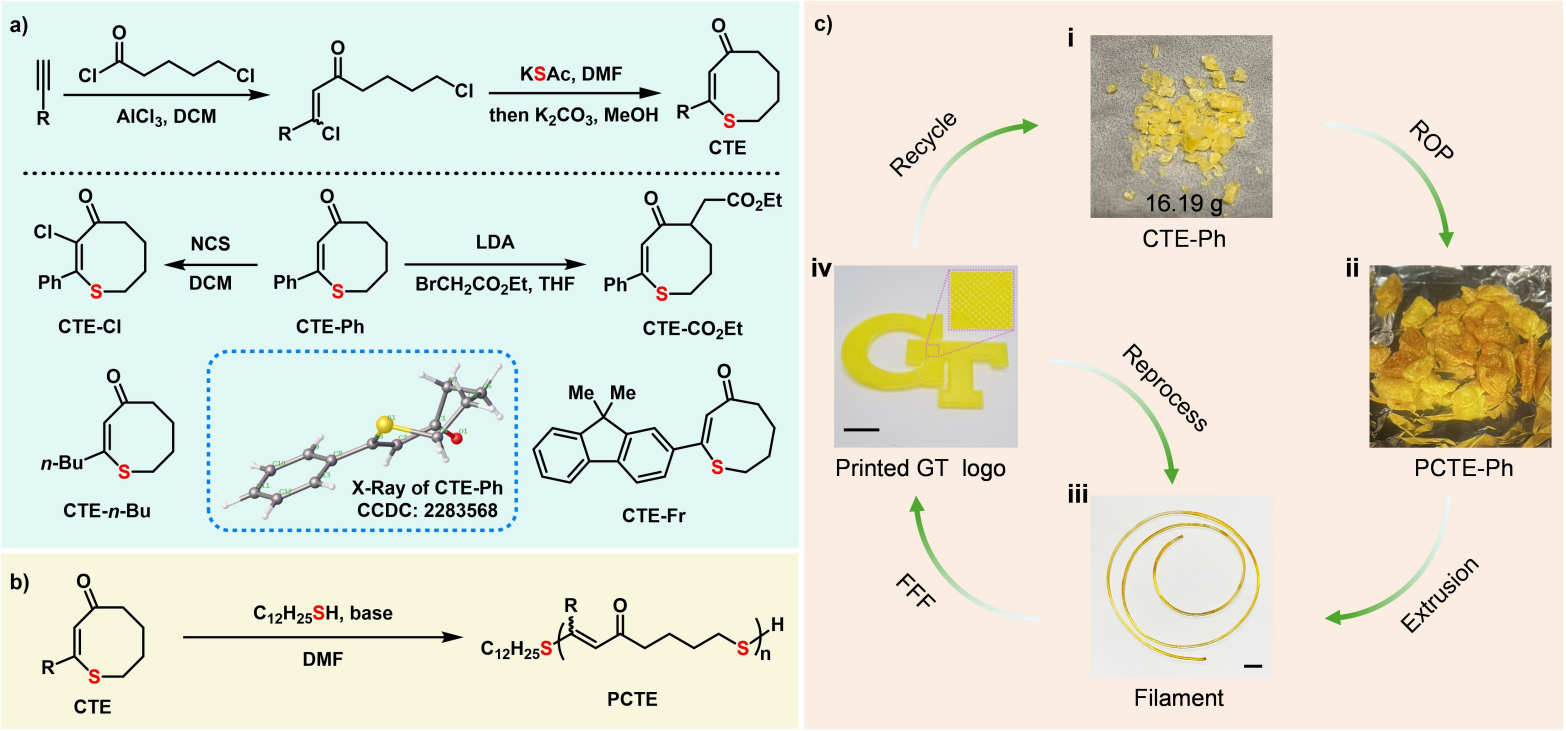
Development and application of PCTE: from synthesis to recycling. **a**, Synthesis of cyclic thioenone (CTE) monomers. The molecular structure of CTE−Ph as determined by SC‐XRD is shown as the insert. **b**, Preparation of PCTE via Michael addition‐elimination ring‐opening polymerization (MAEROP). **c**, Schematic illustration depicting the additive manufacturing process and the mechanical and chemical recycling pathways for PCTE−Ph. All scale bars: 1 cm. Reproduced with permission from Georgia Institute of Technology. Copyright Georgia Institute of Technology, Atlanta, Georgia.

The ceiling temperature (*T*_c_) is a critical parameter in designing chemically recyclable polymers and can be determined using the Gibbs free energy equation: Δ$G_{p}$ =Δ$H_{p}$ ‐*T*Δ$S_{p}$ , where Δ$H_{p}$ represents the change in enthalpy and Δ$S_{p}$ the change in entropy during polymerization. The *T*_c_ signifies the balance point at which the rates of polymerization and depolymerization are equal. The monomer structure significantly affects recycling efficiency by dictating Δ$H_{p}$ and Δ$S_{p}$ , while also playing a vital role in shaping the material‘s mechanical and thermal properties. In light of these factors, eight‐membered CTEs were specifically designed to strike the correct balance of enthalpy (Δ$H_{p}$ ) and entropy (Δ$S_{p}$ ) to enable both polymerization and depolymerization depending on the external conditions (temperature and concentration),^[53]^ and enhance the overall recyclability of the material. Friedel–Crafts acylation reactions between readily accessible alkynes and 5‐chlorovaleroyl chloride provided *β*, δ’‐dichloro vinyl ketones,^[54]^ which subsequently underwent tandem C(sp^3^)–S and C(sp^2^)–S bond formations to construct the desired monomer skeleton upon treatment with potassium thioacetate (Figure 1a). Following this general procedure, the monomers CTE−Ph, CTE‐*n*‐Bu and CTE−Fr were prepared that contained phenyl, *n*‐butyl, and fluorene sidechains, respectively. Furthermore, the presence of carbonyl and C=C groups in CTE−Ph enabled facile *α*‐functionalization through deprotonation with lithium diisopropylamide (LDA), followed by a nucleophilic reaction with ethyl bromoacetate or chlorination with *N*‐chlorosuccinimide (NCS), yielding CTE−CO_2_Et and CTE−Cl monomers, respectively. These monomers were fully characterized with ^1^H NMR, ^13^C NMR and mass spectrometry. Single‐crystal X‐ray diffraction (SC‐XRD) analysis confirmed the ring structure and revealed that the CTE−Ph monomer features an eight‐membered ring in a twist chair‐chair conformation.^[32]^

Initial tests to assess the polymerizability of CTE−Ph were performed using C_12_H_25_SH as the initiator in conjunction with various bases and solvents (Supporting Information Table S1). When 1,8‐diazabicyclo [5.4.0]‐undec‐7‐ene (DBU, p*K*_a_ of the conjugate acid in acetonitrile=24.34) was used as the base, only moderate conversion was observed in the polar aprotic solvent DMF, with even lower conversion measured in less polar solvents such as THF, 1,4‐dioxane and toluene. Further optimization revealed that using a strong base with a lower *p*K_b_ was beneficial for polymerization. Specifically, when *tert*‐butylimino‐tri(pyrrolidino)phosphorane (BTPP, p*K*_a_ of the conjugate acid in acetonitrile=28) was used as the base, full conversion was observed in just 30 seconds when targeting a degree of polymerization (DP, [M_0_] : [I_0_]) of 20 (Table 1, entry 1). By varying the monomer to initiator ratio, a series of PCTE−Ph with tunable molecular weights was obtained (Table 1, entries 2–5, Figure 2a). Notably, the molecular weight (*M*_n_) exhibited a linear increase, reaching 226.8 kDa when a DP of 400 was targeted. After terminating a polymerization (DP20) with iodoacetamide, the end‐functionalized PCTE−Ph was analyzed by matrix‐assisted laser desorption/ionization‐time‐of‐flight (MALDI‐TOF) mass spectrometry (Figure 2c). The major group of molecular ion peaks were separated by 218.3 Da, which is consistent with the molar mass of the CTE−Ph monomer. Detailed analysis of each signal confirmed the linear structure of PCTE−Ph with (C_12_H_25_S−/−CH_2_CONH_2_) chain‐end groups, validating the fidelity of the chain ends and the high efficiency of the end‐capping process. Next, other CTE monomers were subjected to the optimized ROP conditions, yielding various PCTE polymers (Table 1, entries 6–9; Supplementary Figures 2–5). While CTE‐*n*‐Bu and CTE‐CO_2_Et achieved high conversions during polymerization, CTE−Cl and CTE−Fr exhibited lower polymerizability (Supporting Information Table S2), likely due to the reduced monomer solubility, which in turn decreased the initial monomer concentration. The produced polythioenones exhibited high thermal stability, with glass transition ranging from −23 °C to 79 °C, as determined by thermogravimetric analysis (TGA) and differential scanning calorimetry (DSC) (Figure S7). In addition, the seven‐membered cyclic thioenone (CTE−Ph‐7) was synthesized but failed to polymerize, likely due to its lower ring strain compared to the eight‐membered ring (Supporting Information Table S3).

**Table 1:** MAEROP Results for cyclic thioenone (CTE) monomers.^[a]^

entry	CTE	base	C (M)	[M]_0_ : [I]_0_ : [base]_0_	T (min)	conv.^[b]^	*M*_n,SEC_ (kDa)^[c]^	*Ð* ^[c]^	*T*_d,5%_ (°C)^[d]^	*T*_g_ (°C)^[e]^
1	CTE−Ph	BTPP	2.0	20 : 1 : 1	0.5	98 %	13.6	1.84	–	–
2	CTE−Ph	BTPP	2.0	50 : 1 : 1	2	96 %	28.6	1.61	285	17
3	CTE−Ph	BTPP	2.0	100 : 1 : 1	15	96 %	56.1	1.71	–	–
4^[f]^	CTE−Ph	BTPP	2.5	200 : 1 : 1	300	>99 %	101.6	1.82	295	24
5	CTE−Ph	BTPP	2.5	400 : 1 : 1	1200	66 %	226.8	1.64	–	–
6	CTE‐*n*‐Bu	BTPP	2.0	50 : 1 : 1	10	92 %	23.3	1.73	306	−23
7	CTE−CO_2_Et	BTPP	2.5	50 : 1 : 1	30	96 %	22.7	1.64	300	31
8^[g]^	CTE−Cl	DBU	1.4	20 : 1 : 1	360	76 %	12.9	1.99	267	14
9^[h]^	CTE−Fr	DBU	1.0	20 : 1 : 1	1320	65 %	6.0	1.98	290	79

[a] Polymerizations were performed under N_2_ atmosphere with C_12_H_25_SH as the initiator. [b] Monomer conversion, determined by ^1^H NMR spectroscopy in CDCl_3_. [c] Number‐average molecular weight (*M*_n,SEC_), and dispersity index (*Ð)*, determined by CHCl_3_ size‐exclusion chromatography (SEC) calibrated using polystyrene standards. [d] Temperature causing a 5 % weight loss, measured by thermogravimetric analysis (TGA). [e] Glass transition temperature, measured by differential scanning calorimetry (DSC). [f] Reaction was repeated three times at 18.0 mmol scale. [g] 40 °C. [h] 70 °C. C (M): initial monomer concentration. [I]: initiator. T: reaction time.

**Figure 2:**
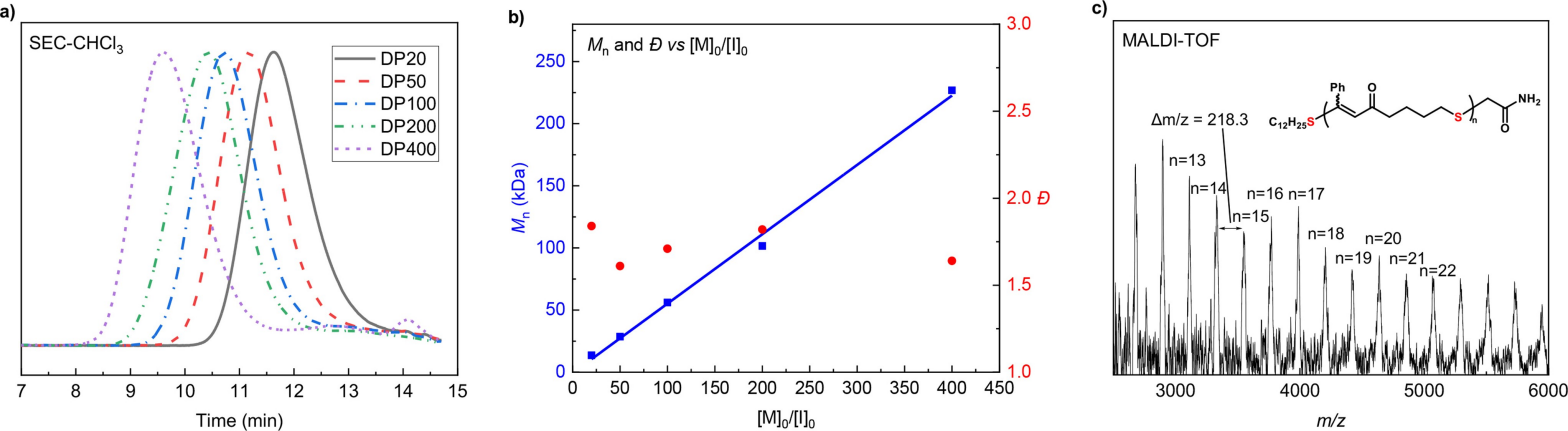
Controlled MAEROP of CTE−Ph and characterization of PCTE−Ph. **a**, SEC curves for PCTE−Ph produced at different [CTE−Ph]_0_ : [BTPP]_0_ : [C_12_H_25_SH]_0_ ratios. **b**, *M*_n_−[M]_0_/[I]_0_ correlation (blue) and *Đ*−[M]_0_/[I]_0_ correlation (red) of MAEROP of CTE−Ph. **c**, MALDI‐TOF mass spectrum of PCTE−Ph (DP20), terminal thiol capped by iodoacetamide.

The scalability of this process was demonstrated by preparing over 16 g of CTE−Ph monomer and 13 g PCTE−Ph. The bulk PCTE−Ph was melt‐pressed between two Teflon sheets to prepare a transparent film with a thickness of 200 μm. This sulfur‐containing polymer, with a brown to yellow color, exhibited a decrease in light transmittance from 70 % at 550 nm to 35 % at 450 nm, while effectively blocking wavelengths below 420 nm (Figure 3a). The material shows promise for use in UV filtering applications as it is capable of blocking nearly 100 % of UV light yet maintaining favorable visible light transmittance.

**Figure 3:**
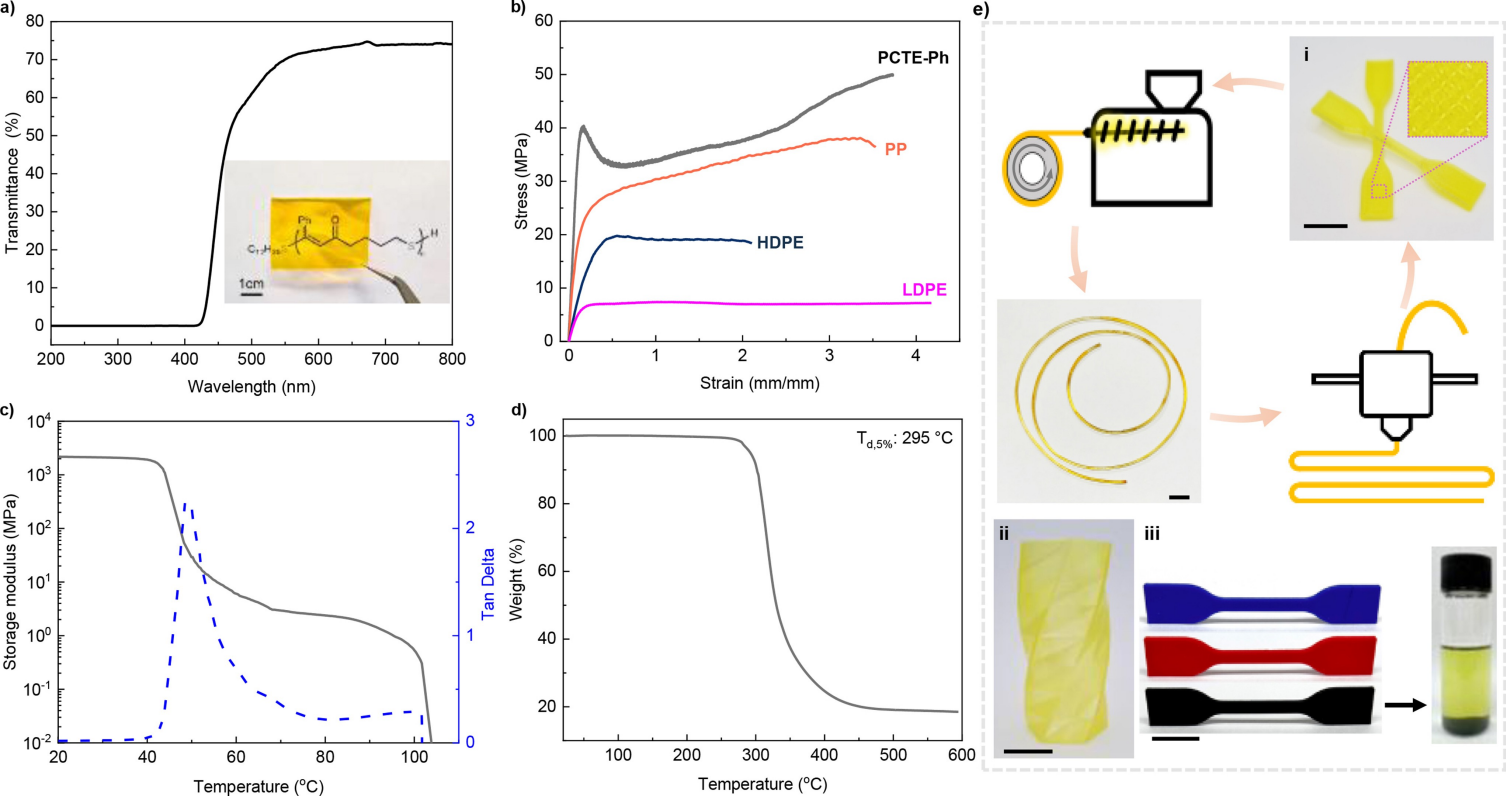
Properties and applications of PCTE−Ph_200_. **a**, UV/Vis transmittance and a photo image of a transparent PCTE−Ph_200_ film over the printed polymer chemical structure. **b**, PCTE−Ph_200_ exhibits superior tensile strength compared to isotactic polypropylene (*i‐*PP), high‐density polyethylene (HDPE) and low‐density polyethylene (LDPE). **c**, DMA storage modulus and tan δ profiles. **d**, PCTE−Ph_200_ shows good thermal stability (T_d,5%_: 295 °C). **e**, PCTE−Ph_200_ was fabricated via fused‐filament fabrication (FFF) 3D printing. The printed prototype items include a vase (ii) and dog bones (i and iii) featuring different colorants or carbon black additives. All scale bars: 1 cm.

The mechanical properties of a thermoplastic typically improve as the polymer molecular weight increases. Here, the bulk sample PCTE−Ph (64.4 kDa) (Figure S8) demonstrated an ultimate tensile strength (σB) of 21.8±0.6 MPa, an elongation at break (ϵΒ) of 130±4.5 %, and a calculated Young's modulus of 455±38 MPa. Increasing the molecular weight to 101.6 kDa resulted in a yield strength increase to 40.4±2.5 MPa, accompanied by pronounced strain hardening behavior and an elongation of 373±19.7 %, highlighting the material‘s exceptional toughness and ductility (Figure 3b). Notably, the tensile strength of PCTE−Ph_200_ (Table 1, entry 4; the subscript “200” indicates the targeted DP) outperforms that of most common commodity plastics, including isotactic polypropylene (*i*‐PP, σB=38±4.8 MPa and ϵΒ=350±8.4 %), HDPE (σB=19.7±2.1 MPa and ϵΒ=210±10.2 %), and LDPE (σB=7.4±1.32 MPa and ϵΒ=410±23 %), thereby suggesting PCTE−Ph as a potential green alternative to these general commodity plastics. Given its high monomer conversion (shown in Table 1) and excellent mechanical performance, PCTE−Ph_200_ was selected for further evaluation to explore its potential in additive manufacturing.

The PCTE−Ph_200_ sample was further analyzed using dynamic mechanical analysis (DMA) in a tensile film mode. Typical thermoplastic behavior with two‐stage dropping of storage modulus was observed (Figure 3c). The first stage, associated with the glass transition temperature, was determined by the tan δ peak at 49 °C. The second sharp drop in storage modulus around 100 °C is attributed to the flow of PCTE−Ph. The low flow temperature facilitates easy processing at relatively low temperatures, which is advantageous for thermal processing techniques. The 5 % decomposition temperature of PCTE−Ph_200_ is 295 °C, as determined by thermogravimetric analysis, significantly higher than its melting processing temperature, thus avoiding thermal degradation during thermal reprocessing (Figure 3d).

Furthermore, as part of a demanding processing approach, additive manufacturing via fused‐filament fabrication (FFF) was explored. As schematically presented in Figure 3e, PCTE−Ph_200_ can be easily extruded into printable filaments, allowing for straightforward printing with any conventional FFF 3D printer. Its low melting point facilitates the recycling and reproduction of the filament via direct extrusion. The PCTE−Ph_200_ filament displayed excellent printability when compared to commercial filaments such as PLA and PVA. Even the single filament‐layered twisted cylinder achieved high shape fidelity and smooth detailed surface. Filaments incorporating different colorants or fillers, as demonstrated with carbon black in Figure 3e, did not compromise printing quality. The filler could be precipitated and separated from the polymer by dissolving in chloroform, with the dissolved PCTE−Ph_200_ recoverable for reprocessing by simply evaporating the solvent.

Given its low flow point compared to its degradation temperature, PCTE−Ph avoids unintended degradation during reprocessing and remains stable through multiple cycles of direct thermal reprocessing, as shown in Figure 4a and b. Both mechanical and thermal mechanical properties remained within a range comparable to the virgin sample.

**Figure 4:**
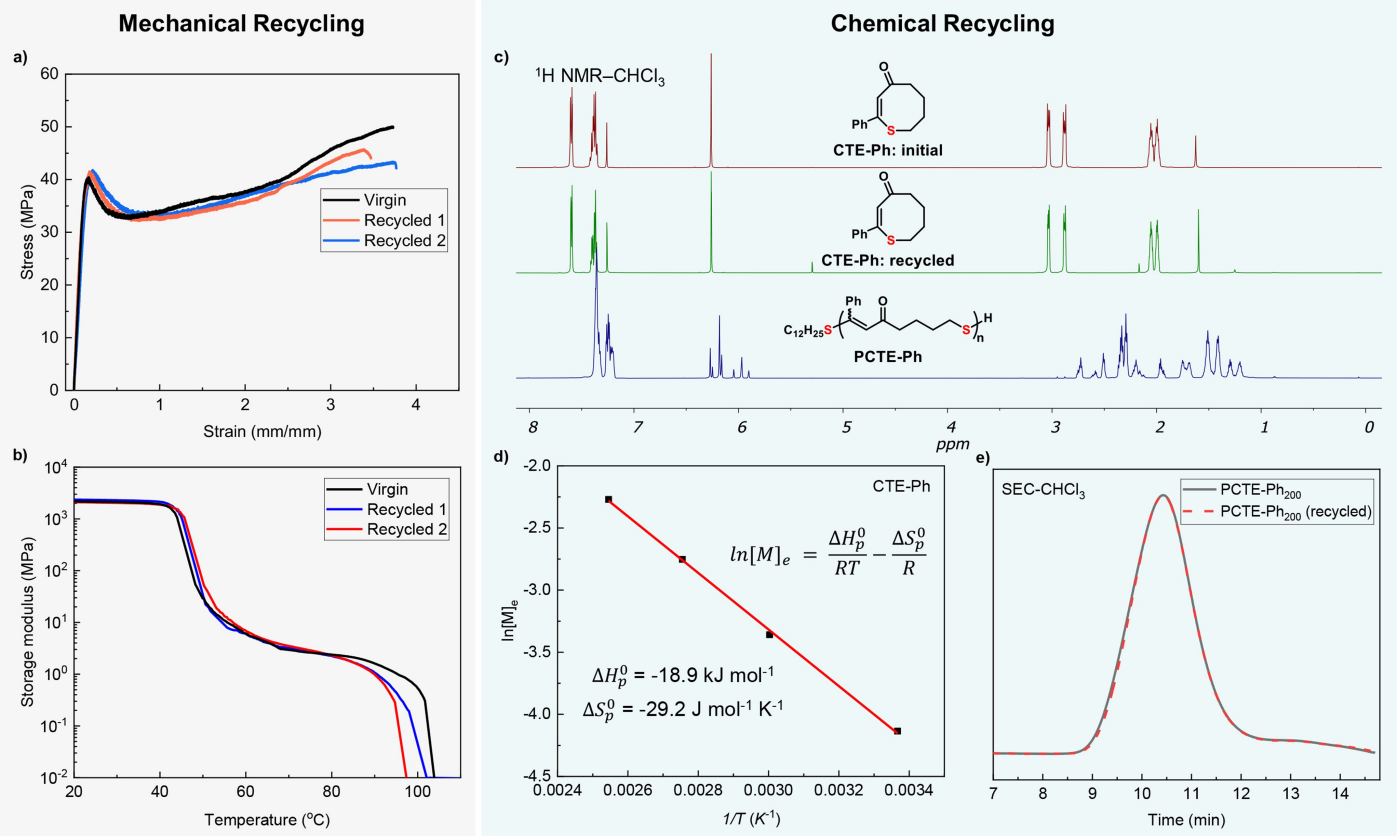
Mechanical recyclability and chemical recyclability of PCTE−Ph_200_. **a**,**b**, PCTE−Ph maintains consistent tensile strength (**a**) and storage modulus (**b**) after mechanical recycling. **c**, Overlay of ^1^H NMR spectra measured in CDCl_3_ of initial CTE−Ph monomer, recycled CTE−Ph and PCTE−Ph. **d**, The van't Hoff plot of CTE−Ph ([M]_0_=0.25 M), the enthalpy change ${\Delta H}_{p}^{0}$ and entropy change ${\Delta S}_{p}^{0}$ were calculated from the slope and intercept of the plot. **e**, SEC traces of PCTE−Ph_200_ prepared from pristine monomer (black line) and recycled monomer (red line).

In addition to reprocessing capabilities, PCTE−Ph can also undergo catalytic depolymerization to recover the virgin quality CTE monomer. A 90 % yield of the monomer CTE−Ph was successfully isolated using substoichiometric equivalents of DBU and C_12_H_25_SH at a concentration of 10 mg/mL and a reaction temperature of 150 °C in DMF (Figure 4c, Supporting Information Tables S4–5). Thermodynamic analysis of the ROP with pristine CTE−Ph was performed, as shown in Figure 4d. The thermodynamic parameters were derived through linear regression of the plot of ln[M]_e_ against 1/T according to the van't Hoff equation, where [M]_e_ represents the equilibrium monomer concentration. The calculated enthalpy change $\left(\Delta H_{p}^{0}\right)$ was −18.9 kJ mol^−1^, and the entropy change ${(\Delta S}_{p}^{0})\;$ was −29.2 J mol^−1^ K^−1^. Moreover, the recovered CTE−Ph monomer could be used to resynthesize PCTE−Ph with high reproducibility and conversion, thereby demonstrating the robustness of this closed‐loop recycling process (Figure 4e). This ability not only enhances the material's sustainability profile but also underscores the practical feasibility of its recycling pathway. To examine the effect of substituents on depolymerization efficiency, we tested PCTE−CO_2_Et and PCTE−Cl. While PCTE−CO_2_Et exhibited a high recovery yield (89 %), PCTE−Cl yielded only 20 % (Supplementary Figure 9). The reduced efficiency with PCTE−Cl is attributed to the influence of the Cl substituents, which may affect the depolymerization process.

In conclusion, a new platform for reprocessable and recyclable materials was successfully developed by merging reversible thia‐Michael addition‐elimination with ring‐opening polymerization. The designed monomer was readily synthesized, scalable, and easily modified, enabling fine‐tuning of the material's properties. The resulting high molecular weight PCTE−Ph is a thermally stable thermoplastics with high tensile strength, and excellent machinability, making it applicable for FFF 3D printing. In addition to the desirable merits of reprocessing, PCTE−Ph exhibits a moderate ceiling temperature, and can be efficiently depolymerized back to its original monomers with a 90 % yield, facilitating a closed‐loop recycling process. This new monomer design strategy represents a promising approach for the development of advanced sustainable materials.

## Conflict of Interests

The authors declare no conflict of interest.

## Supporting information

As a service to our authors and readers, this journal provides supporting information supplied by the authors. Such materials are peer reviewed and may be re‐organized for online delivery, but are not copy‐edited or typeset. Technical support issues arising from supporting information (other than missing files) should be addressed to the authors.

Supporting Information

## Data Availability

The data that support the findings of this study are available from the corresponding author upon reasonable request.
