# Supplementary material for: Reprocessable and Recyclable Materials for 3D Printing via Reversible Thia‐Michael Reactions
Source: Angew Chem Int Ed Engl. 2025 Jan 28;64(8):e202423522. doi: 10.1002/anie.202423522 (PMC11833278; doi:10.1002/anie.202423522)
Supplement: Supplementary file 1 — Supporting Information [file ANIE-64-e202423522-s001.pdf]

Angewandte Chemie
Eine Zeitschrift der Gesellschaft Deutscher Chemiker

Supporting Information

Reprocessable and Recyclable Materials for 3D Printing via Reversible Thia-Michael Reactions

Y.-L. Su, L. Yue, M. K. Paul, J. Kern, K. S. Otte, R. Ramprasad, H. J. Qi, W. R. Gutekunst**

Supporting Information

Reprocessable and Recyclable Materials for 3D Printing via Reversible Thia-Michael Reactions

Yong-Liang Su[1,†], Liang Yue[2,†], McKinley K. Paul[1], Joseph Kern[3], Kaitlyn S. Otte[1], Rampi Ramprasad[3], H. Jerry Qi[2,*] and Will R. Gutekunst[1,*]

[1]*School of Chemistry and Biochemistry, Georgia Institute of Technology, 901 Atlantic Drive NW, Atlanta, Georgia 30332, United States.*
[2]*School of Mechanical Engineering, Georgia Institute of Technology, Atlanta, Georgia 30332, United States*
[3]*School of Materials Science and Engineering, Georgia Institute of Technology, Atlanta, Georgia 30332, United States*

[†]These authors contributed equally to this work.
[*]Correspondence to: qih@me.gatech.edu; willgute@gatech.edu

Table of Contents

1. General information S2
2. Synthesis of cyclic thioenone (CTE) monomers S2
3. Optimization of the polymerization conditions S5
4. General polymerization procedure and characterization of polymers S6
5. Characterizing polymer chain-ends using MALDI-TOF S9
6. TGA and DSC Studies of PCTE S10
7. Mechanical and optical properties of PCTE-Ph S10
8. Chemical recycling of PCTE-Ph S11
9. Polymerization thermodynamic studies of CTE-Ph S13
10. X-ray crystal structure of the monomer CTE-Ph S15
11. NMR spectrum of products S17
12. References S27

1. General information

General methods. All reactions were carried out under a nitrogen atmosphere with dry solvents using anhydrous conditions unless otherwise stated. Dry, degassed *N*,*N*-dimethylformamide (DMF), acetonitrile (CH_3CN), and tetrahydrofuran (THF) were obtained from a JC Meyer solvent purification system. Dimethyl sulfoxide (DMSO), dimethylacetamide (DMA), *N*-methyl-2-pyrrolidone (NMP), *N*,*N′*-dimethylpropyleneurea (DMPU), nitrobenzene ($PhNO_2$), chloroform ($CHCl_3$) and 1,2-dichloroethane (DCE) were purchased from commercial sources and further dried with activated 4Å molecular sieves and degassed. The compounds alkynes, aluminum chloride ($AlCl_3$), 5-chloropentanoyl chloride, potassium thioacetate (KSAc), and ethyl bromoacetate were purchased from TCI. Lithium diisopropylamide (LDA), HPDE (product no.: 427985), LDPE (product no.: 428043) and *i*-PP (product no.: 427888) were purchased from Sigma-Aldrich. *N*-Chlorosuccinimide, other general reagents and solvents were purchased from Fisher Scientific. Yields refer to chromatographically and spectroscopically (^{1}H-NMR) homogeneous materials, unless otherwise stated. Reactions were monitored by thin layer chromatography (TLC) carried out on 0.25 mm E. Merck silica gel plates (60F-254) using UV light as the visualizing agent and basic aqueous potassium permanganate ($KMnO_4$), and heat as developing agents. E. Merck silica gel (60, particle size 0.043–0.063 mm) was used for flash column chromatography. NMR spectra were recorded on Bruker Avance 400, 500 or 700 MHz instruments and calibrated using residual undeuterated solvent as an internal reference ($CHCl_3$ @ 7.26 ppm ^{1}H NMR, 77.16 ppm ^{13}C NMR). The following abbreviations (or combinations thereof) were used to explain the multiplicities: s = singlet, d = doublet, t = triplet, q = quartet, m = multiplet, br = broad, comp = composite of magnetically non-equivalent protons. Mass spectra (MS) were recorded on LC/MS (Agilent Technologies 1260 Infinity II/6120 Quadrupole) or a time-of-flight matrix assisted laser desorption/ionization (MALDI-TOF) using a *trans*-2-[3-(4-*tert*-butylphenyl)-2-methyl-2-propenylidene]malononitrile (DCTB) matrix. Polymer samples were analyzed using a Tosoh EcoSEC HLC 8320GPC system with TSKgel SuperHZ-L columns eluting $CHCl_3$ containing 0.25% NEt_3 at a flow rate of 0.45 mL/min. All number-average molecular weights and dispersities were calculated from refractive index chromatograms using PStQuick Mp-M polystyrene standards. Thermogravimetric analyses (TGA) were performed under nitrogen atmosphere on a Pyris 1 TGA (PerkinElmer) at a heating rate of 10 °C/min. Differential scanning calorimetry (DSC) analyses were measured on a DSC 3+ STARe system (Mettler Toledo). The reported data were obtained from the third heating cycle at a heating rate of 10 °C/min. Melting points were measured on a MEL-TEMP II Laboratory Devices (uncorrected).

2. Synthesis of cyclic thioenone (CTE) monomers

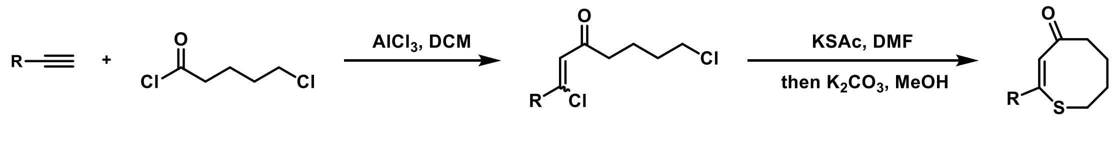

The monomer CTE-Ph, CTE-*n*-Bu and CTE-Fr were prepared according to the procedures reported in the literature with some modifications.[1]

Representative procedure for Friedel-Crafts acylation of alkynes:

To a stirred suspension of aluminum chloride (73.3 g, 550 mmol) in dry dichloromethane (250 mL) at 0 °C, was added a mixture of phenylacetylene (51.05 g, 54.9 mL, 500 mmol) and 5-chloropentanoyl chloride (77.5 g, 64.3 mL, 500 mmol) dropwise over 30 min. The cooling bath was removed, and the reaction mixture was warmed up to ambient temperature over one hour. The mixture was poured into a mixture of brine and ice. The mixture was stirred for two hours at ambient temperature and extracted using dichloromethane (3×200 mL). The combined organic layers were washed with brine, dried over magnesium sulfate and concentrated in vacuo. The residue was purified by column chromatography on silica gel (eluent: hexanes/ diethyl ether = 10:1) to give the alkene product (pale yellow solid, 70% yield, 90.1 g). The spectral data were in accordance with those reported in the literature.[1]

Modified procedure for cyclization with KSAc:

To a stirred solution of (*Z*)-1,7-dichloro-1-phenylhept-1-en-3-one (90.1 g, 350.3 mmol) in DMF (1750 mL, 0.2 M) was added potassium thioacetate (KSAc, 525.5 mmol, 60.0 g) at ambient temperature. The reaction was complete in 5 h as evidenced by thin layer chromatography. The reaction mixture was diluted with H_2O (200 mL), then extracted with ethyl acetate (3×200 mL). The combined organic layers were washed with brine, dried over magnesium sulfate and concentrated in vacuo. The crude product was used in next step without purification.

To a stirred solution of above obtained crude product (44.9 g, 151.4 mmol) in MeOH (3000 mL, 0.05 M) was added potassium carbonate (K_2CO_3, 23.0 g, 166.5 mmol) at 0 °C. The reaction was allowed to warm up to ambient temperature slowly and stirred for an additional 5 h. The mixture was then quenched with HCl (2 M, 170 mL), and the MeOH was removed by evaporation. The resulting mixture was extracted with ethyl acetate (3×200 mL) and the combined organic layers were washed with brine, dried over magnesium sulfate and concentrated in vacuo. The residue was purified by column chromatography on silica gel (eluent: hexanes/ ethyl acetate = 12:1) to give the cyclic thioenone monomer CTE-Ph (pale yellow solid, 49% yield in two steps, 16.2 g). The spectral data were in accordance with those reported in the literature.[1]

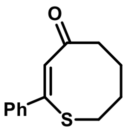

CTE-Ph

(*Z*)-2-Phenyl-5,6,7,8-tetrahydro-4*H*-thiocin-4-one.
^{1}H NMR (500 MHz, chloroform-*d*) δ 7.65 – 7.57 (comp, 2H), 7.50 – 7.31 (comp, 3H), 6.26 (s, 1H), 3.12 – 2.97 (m, 2H), 2.97 – 2.81 (m, 2H), 2.12 – 1.92 (comp, 4H). **^{13}C NMR** (176 MHz, chloroform-*d*) δ 205.9, 154.3, 140.1, 130.1, 128.6, 128.2, 124.8, 41.0, 34.4, 29.9, 22.1. **MS (m/z)**: calcd for $C_{13}H_{15}OS$, $[M+H]^+$: 219.08; found, 219.2.

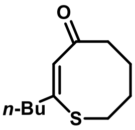

CTE-*n*-Bu

(*Z*)-2-Butyl-5,6,7,8-tetrahydro-4*H*-thiocin-4-one.
^{1}H NMR (500 MHz, chloroform-*d*) δ 5.94 (s, 1H), 2.90 – 2.82 (m, 2H), 2.75 – 2.65 (m, 2H), 2.34 – 2.23 (m, 2H), 2.06 – 1.96 (m, 2H), 1.90 (tt, *J* = 8.4, 4.6 Hz, 2H), 1.62 – 1.51 (m, 2H), 1.39 – 1.30 (m, 2H), 0.92 (t, *J* = 7.4 Hz, 3H). **^{13}C NMR** (176 MHz, chloroform-*d*) δ 207.0, 154.7, 123.1, 42.4, 40.2, 32.9, 31.8, 30.5, 22.2, 21.6, 14.0. Peak overlapping was observed. **MS (m/z)**: calcd for $C_{11}H_{19}OS$, $[M+H]^+$: 199.12; found, 199.1. Overall three-step yield: 28%. The spectral data were in accordance with those reported in the literature.[1]

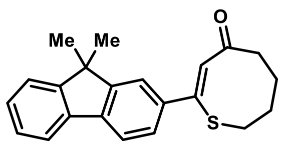

CTE-Fr

(*Z*)-2-(9,9-dimethyl-9*H*-fluoren-2-yl)-5,6,7,8-tetrahydro-4*H*-thiocin-4-one.
^{1}H NMR (500 MHz, chloroform-*d*) δ 7.78 – 7.59 (comp, 4H), 7.45 (dd, *J* = 5.9, 2.7 Hz, 1H), 7.39 – 7.31 (comp, 2H), 6.38 (s, 1H), 3.15 – 3.04 (m, 2H), 2.99 – 2.89 (m, 2H), 2.12 – 1.97 (comp, 4H), 1.50 (s, 6H).**^{13}C NMR** (176 MHz, chloroform-*d*) δ 205.7, 155.6, 154.3, 154.0, 141.6, 139.0, 138.4, 128.1, 127.6, 127.3, 124.8, 122.8, 122.6, 120.6, 120.0, 47.1, 40.5, 34.9, 29.6, 27.2, 22.3. Overall three-step yield: 5%. Peak overlapping was observed. **MS (m/z)**: calcd for $C_{22}H_{23}OS$, $[M+H]^+$: 335.15; found, 335.2.

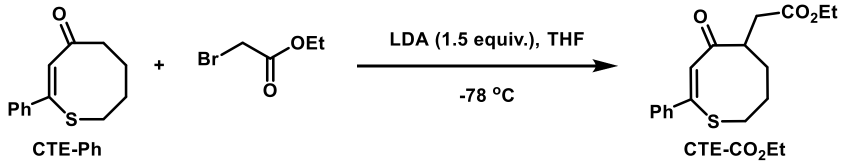

To a flask equipped with a stirring bar were added **CTE-Ph** (0.66 g, 3 mmol, 1.0 equiv.), anhydrous THF (20 mL), and the system was cooled to -78 °C. Lithium diisopropylamide (LDA, 2 M, 2.4 mL, 4.8 mmol, 1.6 equiv.) was then added dropwise, and the reaction mixture was stirred for 1 h. A solution of ethyl 2-bromoacetate (0.75 g, 4.5 mmol, 1.5 equiv.) in anhydrous THF (10 mL) was added dropwise to the reaction mixture. After stirring at -78 °C for 3 h, the reaction was quenched with saturated ammonium chloride solution (10 mL) and allowed to warm gradually toroom temperature. The mixture was extracted with ethyl acetate (3×30 mL), and the combined organic layers were washed with brine, dried over anhydrous Na_2SO_4 and concentrated in vacuo. The crude product was purified by column chromatography on silica gel (eluent: hexane/diethyl ether = 10:1) to give product CTE-CO_2Et (colorless oil, 61% yield, 0.55 g).

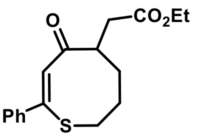

CTE-CO_2Et

Ethyl (*Z*)-2-(6-oxo-8-phenyl-3,4,5,6-tetrahydro-2*H*-thiocin-5-yl)acetate.
^{1}H NMR (400 MHz, chloroform-*d*) δ 7.70 – 7.56 (comp, 2H), 7.43 – 7.30 (comp, 3H), 6.36 (s, 1H), 4.13 (q, *J* = 7.1 Hz, 2H), 3.90 (tdd, *J* = 8.9, 6.1, 2.7 Hz, 1H), 3.15 (ddd, *J* = 15.0, 6.8, 3.1 Hz, 1H), 2.91 (dd, *J* = 16.4, 8.4 Hz, 1H), 2.81 (ddd, *J* = 15.0, 9.7, 2.6 Hz, 1H), 2.41 (dd, *J* = 16.4, 6.1 Hz, 1H), 2.14 – 1.99 (m, 2H), 1.97 – 1.88 (m, 1H), 1.86 – 1.75 (m, 1H), 1.26 (t, *J* = 7.1 Hz, 3H). **^{13}C NMR** (176 MHz, chloroform-*d*) δ 206.4, 172.2, 151.9, 140.2, 130.1, 128.6, 128.3, 126.0, 60.7, 45.0, 37.8, 34.0, 28.6, 27.6, 14.4. **MS (m/z)**: calcd for $C_{17}H_{21}O_3S$, $[M+H]^+$: 305.12; found, 305.1.

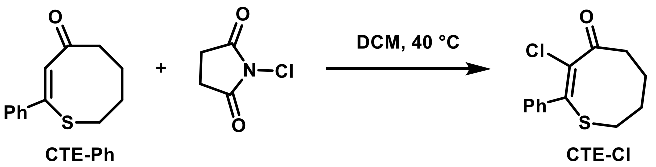

To a flask equipped with a stirring bar were added CTE-Ph (0.66 g, 3 mmol, 1.0 equiv.), *N*-chlorosuccinimide (NCS, 0.48 g, 3.6 mmol, 1.2 equiv.). The reaction mixture was stirred 40 °C for 16 h. After removing the DCM by evaporation, the crude product was purified by column chromatography on silica gel (eluent: hexane/ethyl acetate = 12:1) to give product CTE-Cl (pale yellow solid, 88% yield, 0.67 g).

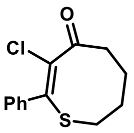

CTE-Cl

(*E*)-3-Chloro-2-phenyl-5,6,7,8-tetrahydro-4*H*-thiocin-4-one.
^{1}H NMR (700 MHz, chloroform-*d*) δ 7.52 – 7.46 (comp, 2H), 7.43 – 7.38 (comp, 3H), 3.04 – 2.94 (comp, 4H), 2.11 – 2.05 (m, 2H), 2.03 – 1.97 (m, 2H). **^{13}C NMR** (176 MHz, chloroform-*d*) δ 201.3, 145.6, 137.6, 129.7, 129.7, 128.4, 121.1, 40.4, 34.6, 29.4, 22.3. **MS (m/z)**: calcd for $C_{13}H_{14}ClOS$, $[M+H]^+$: 253.04; found, 253.1.

3. Optimization of the polymerization conditions

Table S1. Investigation of polymerization conditions of CTE-Ph[a]

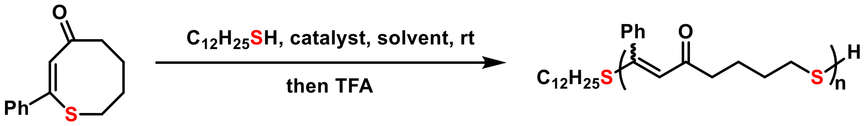

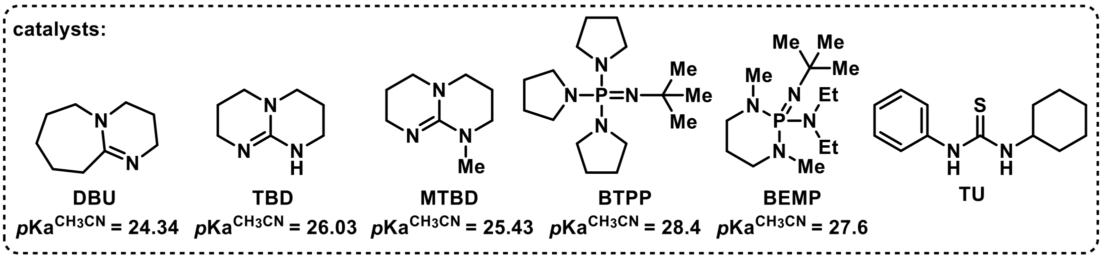

entry	solvent	catalyst	conc. (M)	time	conv. (%)[b]	$M_{n,SEC}$ (kDa)[c]	$Đ$[c]
1	THF/1,4-Dioxane (1/1)	DBU	1.5	22 h	<5	-	-
2	DMF	DBU	1.5	23 h	49	6.9	2.22
3	Toluene	DBU/TU-1	1.5	24 h	<10	-	-
4	Toluene	TBD	1.5	24 h	25	-	-
5	Toluene	TBD/TU-1	1.5	24 h	62	14.4	1.67
6	Toluene	MTBD	1.5	48 h	<5	-	-
7	Toluene	MTBD/TU-1	1.5	48 h	40	8.4	1.60
8	Toluene	BTPP	1.5	24 h	56	14.3	1.58
9	Toluene	BTPP/TU-1	1.5	7 h	90	18.8	1.80
10	THF	BTPP	1.5	22 h	99	23.8	1.90
11	THF	BTPP/TU-1	1.5	4.5 h	94	20.5	1.75
12	DMF	BTPP	2.0	2 min	>99	28.6	1.61
13	DMF	BEMP	2.0	6 min	87	20.3	1.86
14[d]	DMF	BTPP	2.5	5 h	>99	101.6	1.82

[a]$[M]_0/[I]_0/[catalyst]_0$ = 50/1/1. [b]Conversions were determined by ^{1}H NMR spectroscopic analysis of the reaction mixture. [c]Molecular weights ($M_{n,SEC}$)and dispersities ($Đ$)were determined by size-exclusion chromatography. [d]The reaction was repeated three times at 18.0 mmol scale, targeting DP200.

Table S2. Investigation of polymerization conditions of CTE-Cl[a]

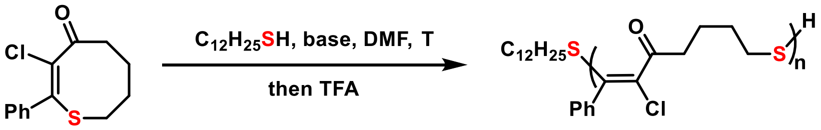

entry	base	temp. (°C)	conc. (M)	time	conv. (%)[b]	$M_{n,SEC}$ (kDa)[c]	$Đ$[c]
1	BTPP	rt	1.0	16 h	15	2.4	2.32
2	BTPP	50	1.25	24 h	17	-	-
3	TBD	50	1.25	21 h	42	7.6	2.69
4	DBU	50	1.0	15 h	33	9.1	1.64
5[d]	DBU	40	1.4	6 h	76	12.9	1.99

[a] $[M]_0/[I]_0/[base]_0 = 50/1/1$. [b] Conversions were determined by ^1H NMR spectroscopic analysis of the reaction mixture. [c] Molecular weights ($M_{n,SEC}$)and dispersities ($Đ$)were determined by size-exclusion chromatography. [d] $[M]_0/[I]_0/[base]_0 = 20/1/1$.

Table S3. Investigation of the polymerization of **CTE-Ph-7**[a]

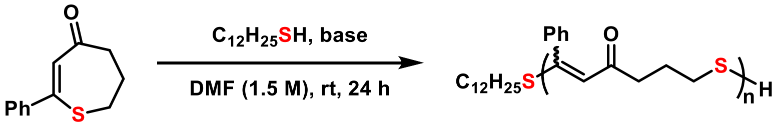

CTE-Ph-7

entry	base	conv. (%)[b]
1	DBU	<5%
2	BTPP	<5%

[a] $[M]_0/[I]_0/[base]_0 = 50/1/1$. [b] Conversions were determined by ^1H NMR spectroscopic analysis of the reaction mixture.

4. General polymerization procedure and characterization of polymers

Preparation of stock solution: The desired amounts of 1-dodencanethiol and BTPP were added into an oven-dried 2 mL vial under N_2. Dry, degassed DMF was then added to make a stock solution. To an oven-dried microwave vial equipped with a magnetic stir bar was added the monomer CTE (0.2 mmol). Following the evacuation and triple backfilling with N_2, the vial was charged with dry, degassed DMF, along with the initiator stock solution. After stirring for the indicated time, the reaction was quenched by three drops of trifluoracetic acid. An aliquot of the reaction mixture was taken for ^1H NMR to determine the conversion of the monomer. Another aliquot of the reaction mixture was taken for SEC analysis. The resulting polymer was precipitated from cold MeOH. The purified polymer was then characterized using SEC, ^1H-NMR, ^{13}C-NMR, TGA and DSC.

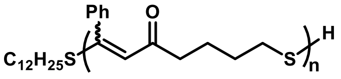

PCTE-Ph

^{1}H NMR (700 MHz, chloroform-*d*) δ 7.41 – 7.30 (comp, 3H), 7.29 – 7.16 (comp, 2H), 6.28 – 5.87 (m, 1H), 2.78 – 2.44 (m, 1H), 2.40 – 2.26 (m, 2H), 2.24 – 1.88 (m, 1H), 1.77 – 1.65 (m, 1H), 1.57 – 1.37 (m, 2H), 1.34 – 1.13 (m, 1H). **^{13}C NMR** (176 MHz, chloroform-*d*) δ 197.9, 160.7, 138.6, 128.8,

128.4, 128.0, 122.8, 42.6, 32.5, 29.2, 23.5. Only the major peaks are listed for reference, as multiple isomers (Z/E) were observed.

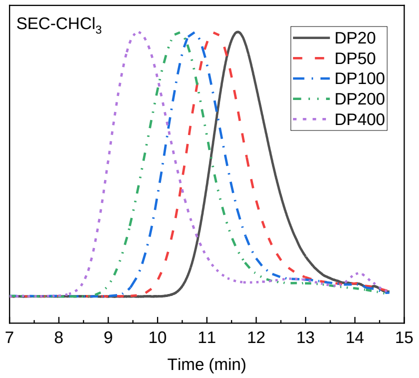

Figure S1. SEC trace for PCTE-Ph targeting different DP.

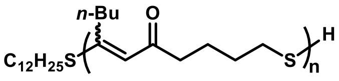

PCTE-*n*-Bu

^{1}H NMR (700 MHz, chloroform-*d*) δ 5.82 (s, 1H), 2.79 – 2.69 (comp, 4H), 2.51 – 2.42 (m, 2H), 1.77 – 1.65 (comp, 4H), 1.57 – 1.47 (m, 2H), 1.42 – 1.32 (m, 2H), 0.92 – 0.89 (m, 3H). **^{13}C NMR** (176 MHz, chloroform-*d*) δ 195.6, 165.6, 115.2, 43.6, 34.9, 32.2, 31.5, 27.2, 23.9, 22.9, 14.0.

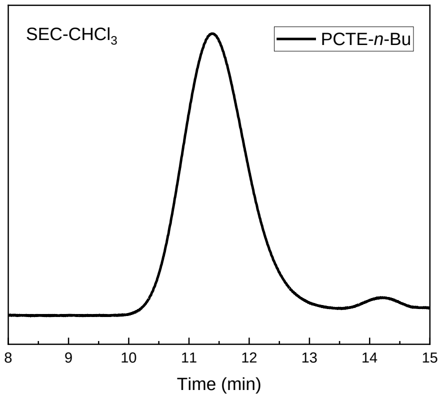

Figure S2. SEC trace of polymer PCTE-*n*-Bu.

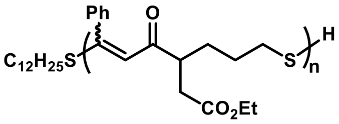

PCTE-CO$_2$Et

^{1}H NMR (700 MHz, chloroform-*d*) δ 7.43 – 7.28 (comp, 3H), 7.25 – 7.16 (comp, 2H), 6.36 – 6.00 (m, 1H), 4.18 – 3.94 (m, 2H), 3.11 – 2.82 (m, 1H), 2.82 – 2.36 (m, 2H), 2.35 – 2.08 (m, 2H), 1.81 – 1.65 (m, 1H), 1.60 – 1.47 (m, 1H), 1.44 – 1.31 (m, 2H), 1.24 – 1.17 (m, 3H). **^{13}C NMR** (176 MHz, chloroform-*d*) δ 199.2, 172.3, 138.6, 129.0, 128.6, 128.3, 128.1, 122.4, 60.7, 47.0, 35.6, 32.7, 31.3, 27.0, 14.3.

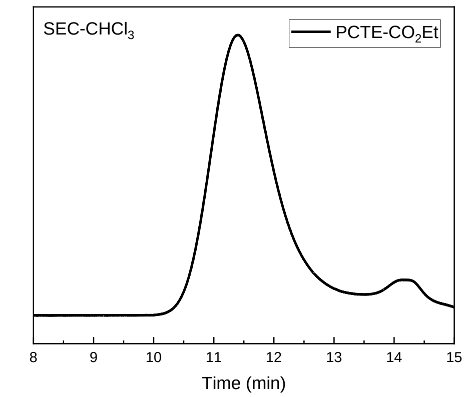

Figure S3. SEC trace of polymer PCTE-CO_2Et.

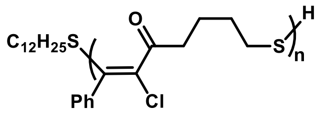

PCTE-Cl

^{1}H NMR (700 MHz, chloroform-*d*) δ 7.54 – 7.32 (comp, 3H), 7.19 – 7.00 (comp, 2H), 2.71 – 2.23 (m, 1H), 2.21 – 1.98 (comp, 3H), 1.63 – 1.49 (m, 1H), 1.33 – 1.15 (comp, 3H). **^{13}C NMR** (176 MHz, chloroform-*d*) δ 193.8, 135.9, 129.1, 128.9, 128.3, 127.8, 123.7, 40.4, 32.1, 28.9, 23.2.

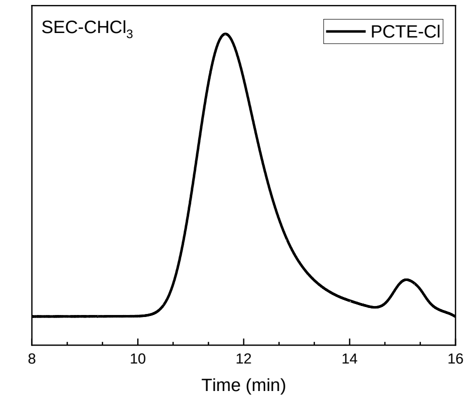

Figure S4. SEC trace of polymer PCTE-Cl.

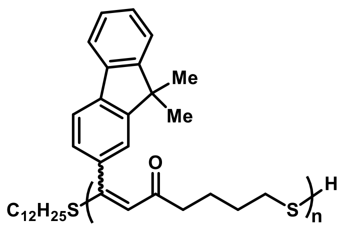

PCTE-Fr

^{1}H NMR (700 MHz, chloroform-*d*) δ 7.76 – 7.57 (comp, 2H), 7.46 – 7.40 (m, 1H), 7.36 – 7.28 (comp, 3H), 7.23 – 7.04 (m, 1H), 6.51 – 5.78 (m, 1H), 2.74 – 2.46 (m, 1H), 2.39 – 2.22 (m, 2H), 1.82 – 1.65 (m, 1H), 1.54 – 1.34 (m, 8H), 1.27 – 1.02 (m, 2H). **^{13}C NMR** (176 MHz, chloroform-*d*) δ 197.8, 161.1, 154.0, 140.1, 138.4, 137.6, 128.0, 127.3, 127.1, 122.8, 122.6, 120.4, 120.4, 120.1, 47.1, 42.7, 32.8, 29.4, 27.2, 23.6. Peak overlapping was observed.

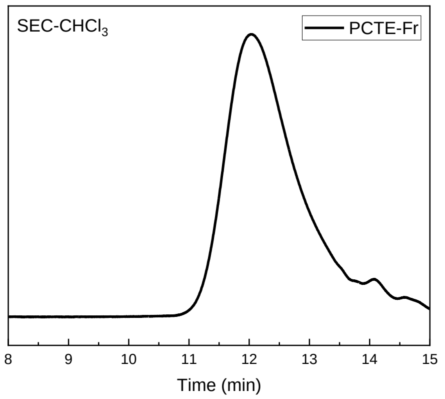

Figure S5. SEC trace of polymer PCTE-Fr.

5. Characterizing polymer chain-ends using MALDI-TOF

Polymerization of CTE-Ph targeting DP20:

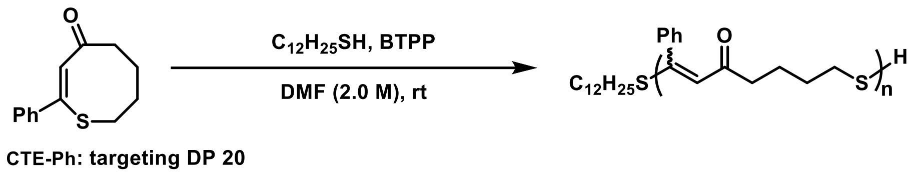

Preparation of stock solution: 1-dodencanethiol (20.24 mg, 24.0 μL, 0.1 mmol) and BTPP (31.24 mg, 30.6 μL, 0.1 mmol) were added into an oven-dried 2 mL vial under N_2. Then dry DMF (445.4 μL) was added to make a stock solution.

To an oven-dried microwave vial equipped with a magnetic stir bar was added the CTE-Ph monomer (0.2 mmol). After evacuation and backfilling with N_2 three times, dry DMF (10 μL) was added followed by the addition of initiator stock solution (50 μL). The total volume is around 100 μL and the initiating concentration of the monomer $[M]_0$ is around 2.0 M. After stirring for 30 seconds, the reaction was quenched with iodoacetamide (100 μL, 1 M). A sample of the crude material was taken for ^{1}H NMR to determine the conversion of the monomer. Another small amount of the crude material was taken for SEC analysis. The product was purified by precipitating from cold methanol (10 mL), then analyzed by matrix-assisted laser desorption/ionization-time-of-flight (MALDI-TOF) mass spectrometry.

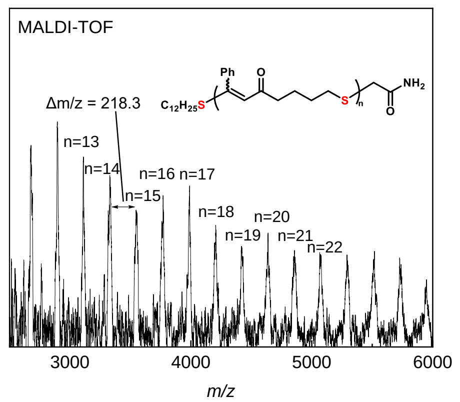

Figure S6. MALDI-TOF of PCTE-Ph (DP20).

6. TGA and DSC Studies of PCTE

TGA of purified PCTEs were obtained in a nitrogen atmosphere at a heating rate of 10 °C/min. The $T_{d,5\%}$ (temperature causing a 5% weight loss) of each PCTE was listed in Figure S9a.
DSC analysis of purified PCTEs were performed (-20 °C to 250 °C or -70 °C to 250 °C, heating rate: 10 °C/min, cooling rate: 10 °C/min). The glass transition temperature (T_g) of each PCTE was listed in Figure S9b.

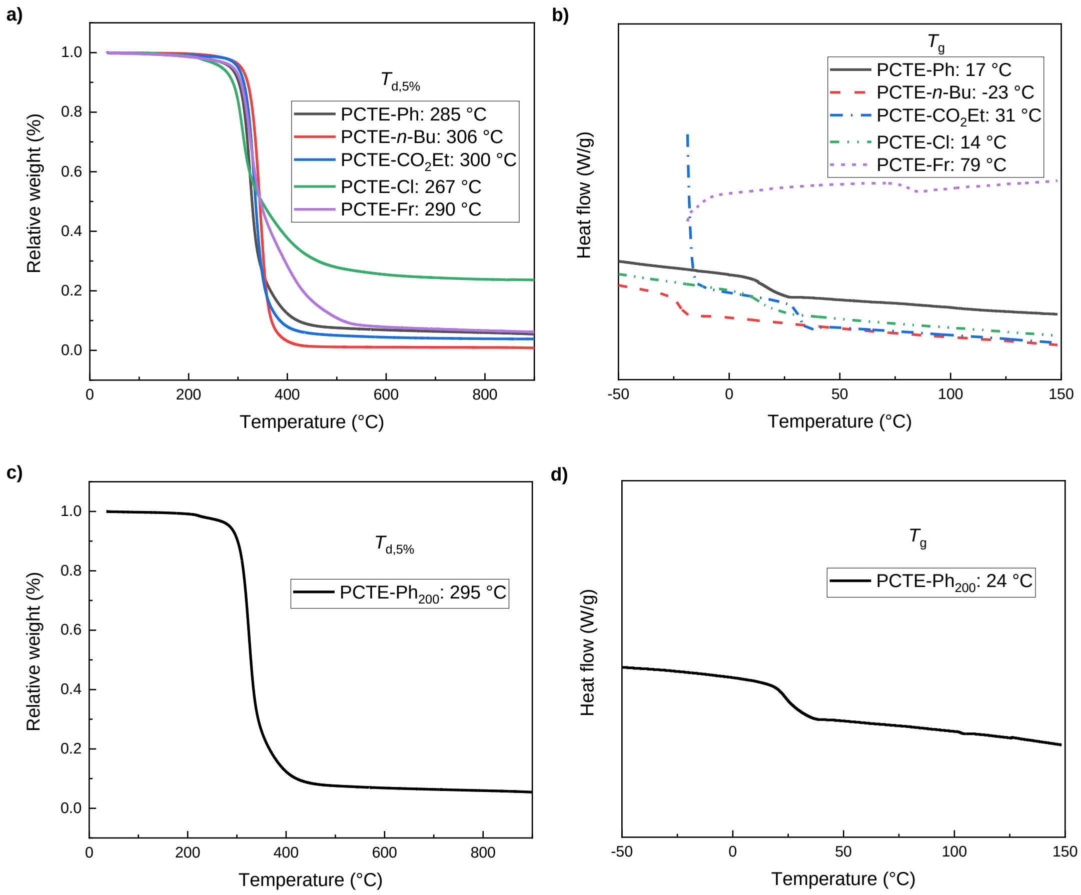

Figure S7. Thermal properties of PCTE. a, TGA curves. b, DSC curves. c, d, thermal properties of PCTE-Ph_{200}.

7. Mechanical and optical properties of PCTE-Ph_{200}

PCTE-Ph_{200} was prepared according to the general polymerization procedure. The polymer was compressed into 0.5mm films at 100 ℃ between two teflon sheets. Then, the sample was cut into strips (5mm x 30mm) for testing. Their thermomechanical properties were measured on a dynamic mechanical analysis tester (Q800, TA Instruments, New Castle, DE) with a frequency of 1 Hz in a tension mode with a temperature ramp 10 ºC/min. The uniaxial tension tests were performed with a universal test machine (Insight 10, MTS Systems Corp., Eden Prairie, MN, USA) with a cross-head speed of 5 mm/min. Multiple tests were conducted for each sample.

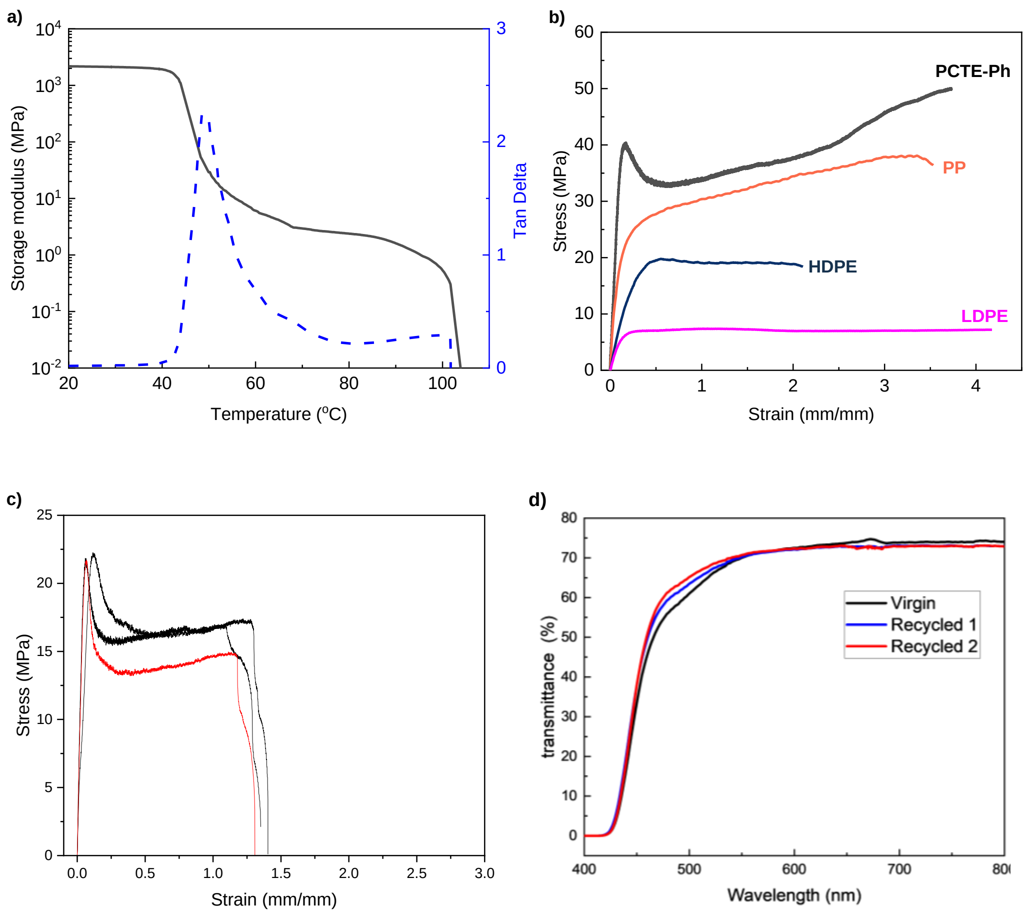

Figure S8. Mechanical properties of PCTE-Ph_{200}. **a**, DMA storage modulus and tanδ profiles of PCTE-Ph_{200}; **b**, Tensile stress-strain curves of PCTE-Ph_{200} (101.6 kDa), PP, HDPE and LDPE. **c**, Tensile stress-strain curves of PCTE-Ph (64.4 kDa). **d**, UV-vis transmittance of PCTE-Ph_{200} after mechanical recycling.

8. Chemical recycling of PCTE

Depolymerization of PCTE-Ph (DP50):

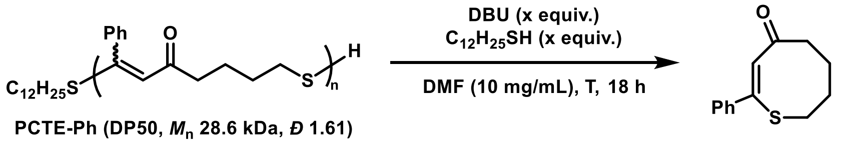

To a solution of PCTE-Ph (DP50, 5.72 mg, 2×10^{-4} mmol, 1.0 equiv.; repeat unit: 1.3×10^{-2} mmol) in DMF was added the stock solution of DBU and $C_{12}H_{25}SH$ in DMF. After stirring for 18 h under the specified temperature, the reaction was quenched by TFA. The solvent was removed by evaporation and the residue was analyzed by ^1H NMR spectroscopy and SEC. The crude mixture from the conditions of 0.5765 equiv. DBU and $C_{12}H_{25}SH$ under 150 °C was purified by then purified by column chromatography on silica gel (eluent: hexanes/ethyl acetate = 12:1) to give product CTE-Ph (92% yield, 5.21 mg).

Table S4. Investigation of the reaction temperature and the amount of DBU and $C_{12}H_{25}SH$ for the depolymerization[a]

entry	temperature (°C)	DBU	$C_{12}H_{25}SH$	conversion (%)[b]
1	90	0.19 equiv.	0.19 equiv.	28%
2	90	0.38 equiv.	0.38equiv.	55%
3	120	0.19 equiv.	0.19 equiv.	34%
4	120	0.38 equiv.	0.38 equiv.	75%
5	150	0.19 equiv.	0.19 equiv.	39%
6	150	0.38 equiv.	0.38 equiv.	81%
7	150	0.58 equiv.	0.58 equiv.	90% (92%)[c]
8	150	0.77 equiv.	0.77 equiv.	78%
9	150	0.96 equiv.	0.96 equiv.	66%

[a]The amount of DBU and $C_{12}H_{25}SH$ was relative to the repeat unit. [b]The conversion was determined by ^{1}H NMR spectroscopic analysis of the reaction mixture. [c]Isolated yield of recovered monomer in parentheses.

Depolymerization of PCTE-Ph (DP200):

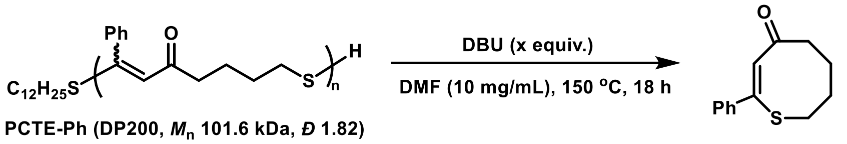

Table S5. Investigation of the depolymerization of PCTE-Ph using DBU alone[a]

entry	DBU	conversion (%)[b]
1	0.19 equiv.	7%
2	0.38 equiv.	17%
3	0.57 equiv.	24%
4	0.76 equiv.	66%
5	0.95 equiv.	43%

[a]The amount of DBU was relative to the repeat unit. [b]The conversion was determined by ^{1}H NMR spectroscopic analysis of the reaction mixture.

Depolymerization of PCTE-Ph (DP200) under optimized reaction conditions:

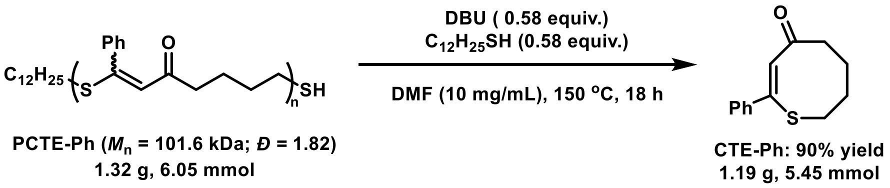

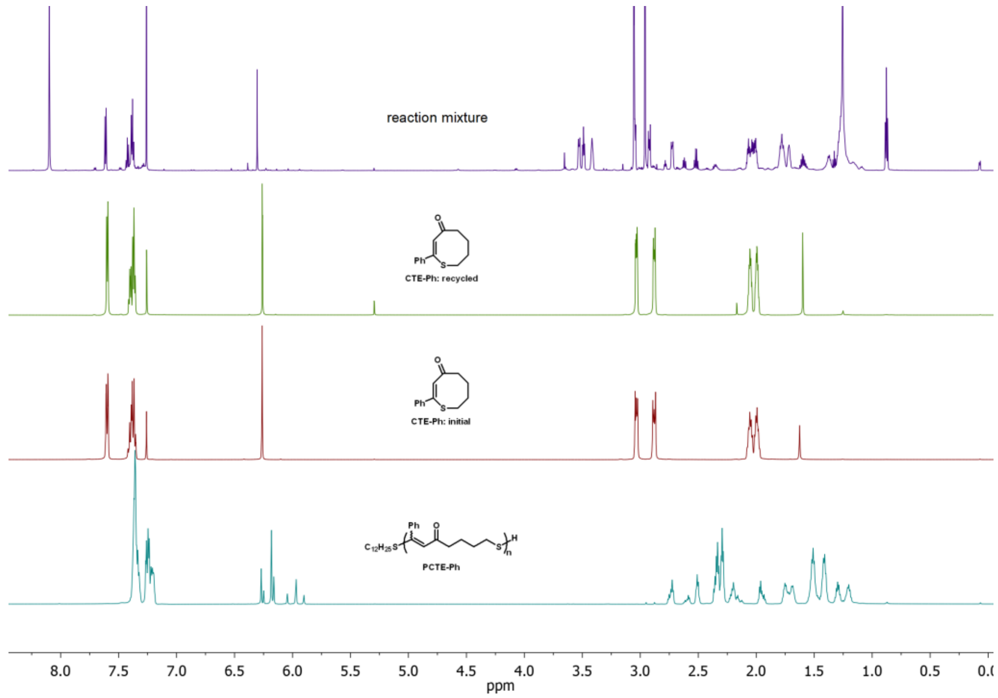

Figure S9. Overlay of ^{1}H-NMR spectra of the reaction mixture under the optimized depolymerization conditions, recycled and initial CTE-Ph and PCTE-Ph.

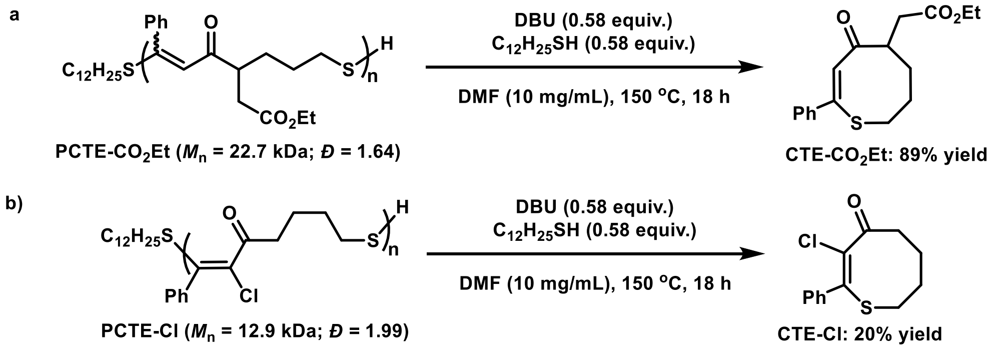

Figure S10. Chemical recycling of PCTE-CO_2Et and PCTE-Cl.

9. Polymerization thermodynamic studies of CTE-Ph

A representative procedure for the polymerization thermodynamic studies is shown as follows. Preparation of stock solution: 1-dodencanethiol (20.24 mg, 24.0 µL, 0.1 mmol) and DBU (15.22 mg, 15.0 µL, 0.1 mmol) were added into an oven-dried 2 mL vial under N_2. Then dry DMSO (211 µL) was added to make a stock solution.

To four oven-dried microwave vials equipped with magnetic stir bars were added the CTE-Ph monomer (0.2 mmol in each vial). After evacuation and backfilling with N_2 three times, 740 µL DMSO was added into each vial. The vials were then placed into a preheated oil bath at different temperatures, including 24 °C, 60 °C, 90 °C and 120 °C. The initiator stock solution (20 µL) was added to the monomer solution. According to kinetic studies, all polymerizations at 24 °C reached equilibrium by 4 h, polymerizations at higher temperatures should require shorter time. After stirring for 4 h, the reaction was quenched by three drops of trifluoracetic acid and cooled to room temperature. The monomer concentration at the equilibrium was determined by ^{1}H NMR.

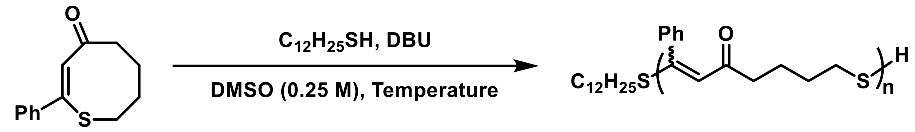

CTE-Ph: targeting DP 25

entry	Temp. (℃)	Temp. (K)	1/T (K^{-1})	conversion (%)	$[M]_0$	$[M]_e$	In $[M]_e$
1	24 ℃	297	0.003367	93.6%	0.25 M	0.016 M	-4.1352
2	60 ℃	333	0.003003	86.1%	0.25 M	0.03745 M	-3.3595
3	90 ℃	363	0.002755	64.5%	0.25 M	0.06375 M	-2.7528
4	120 ℃	393	0.002545	58.7%	0.25 M	0.10325 M	-2.2706

The thermodynamic parameters can be extracted by the linear fitting of the plot of $ln[M]_e$ against *1/T* according to the following equation:

$$ln[M]_e = \frac{\Delta H_p^0}{RT} - \frac{\Delta S_p^0}{R}$$

Here $[M]_e$ is the monomer concentration at thermodynamic equilibrium in mol L^{-1}, T is the reaction temperature in K^{-1}, ΔH_p^0 is the enthalpy change of polymerization in kJ mol^{-1}, ΔS_p^0 is the entropy change of polymerization in J mol^{-1} K^{-1}, and R is the gas constant (8.314 J mol^{-1} K^{-1}).

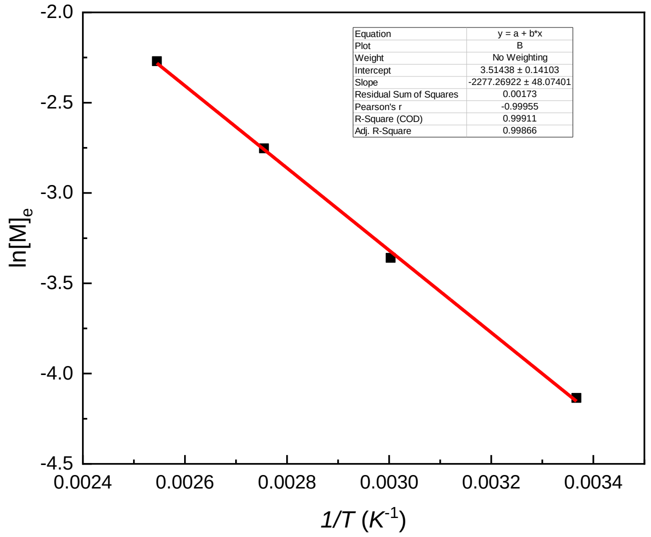

Figure S11. The van 't Hoff plot of CTE-Ph.

$$\Delta H_p^0 = -2277 \times 8.314 \text{ J mol}^{-1} = -18.93 \text{ kJ mol}^{-1}$$

$$\Delta S_p^0 = -3.51 \times 8.314 \text{ J mol}^{-1} \text{ K}^{-1} = -29.18 \text{ J mol}^{-1} \text{ K}^{-1}$$

$$T_c = -18930 \div (-29.18) \text{ K} = 648.7 \text{ K}$$

10. X-ray crystal structure of the monomer CTE-Ph

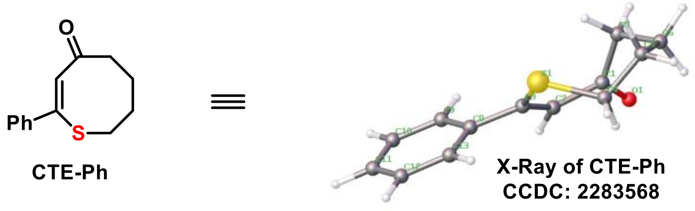

Experimental. Single crystals of $C_{13}H_{14}OS$ CTE-Ph were prepared by slow evaporation of hexane/ethyl acetate solution. Single yellowish triclinic-shaped crystals of CTE-Ph were chosen from the sample. A suitable crystal with dimensions 0.286 × 0.218 × 0.118 mm^3 was selected and mounted on a loop with paratone on a XtaLAB Synergy-S diffractometer. The crystal was kept at a steady T = 100.00 K during data collection. Using Olex 2,[2] The structure was solved with the olex2.solve[3] structure solution program using Charge Flipping and refined with the SHELXL[4] refinement package using Least Squares minimization.

Crystal Data. $C_{13}H_{14}OS$, M_r = 218.32, triclinic, P-1 (No. 2), a = 6.6293(19) Å, b = 9.565(4) Å, c = 10.346(3) Å, α = 62.661(15)°, β = 80.603(11)°, γ = 70.101(12)°, V = 547.9(3) Å^3, T = 100.00 K, Z = 8, μ (Mo K_α) = 0.264 mm^{-1}, 27987 reflections measured, 2000 unique (R_{int} = 0.0987) which were used in all calculations. The final wR_2 was 0.0990 (all data) and R_1 was 0.0414 (I≥2 σ(I)).

Table S6: Crystallographic data and structure refinement for CTE-Ph.

**Compound**	**CTE-Ph**
Formula	$C_{13}H_{14}OS$
$D_{calc.}$/ g cm^{-3}	1.323
μ/mm^{-1}	0.264
Formula Weight	218.32
Color	yellowish
Shape	triclinic-shaped
Size/mm^3	0.286×0.218×0.118
T/K	100.00
Crystal System	triclinic
Space Group	*P*-1
a/Å	6.6293(19)
b/Å	9.565(4)
c/Å	10.346(3)
α/°	62.661(15)
β/°	80.603(11)
γ/°	70.101(12)
V/Å^3	547.9(3)
Z	8
Wavelength/Å	0.71073
Radiation type	Mo K_a
Θ_{min}/°	2.216
Θ_{max}/°	25.33
Measured Refl's.	27987
Indep't Refl's	2000
R_{int}	0.0987
Parameters	137
Restraints	0
Largest Peak	0.26
Deepest Hole	-0.26
GooF	1.074
wR_2 (all data)	0.0990
wR_2	0.0905
R_1 (all data)	0.0576
R_1	0.0414

11. NMR spectrum of products

^{1}H NMR (500 MHz, $CDCl_3$) and ^{13}C NMR (176 MHz, $CDCl_3$) spectra for CTE-Ph

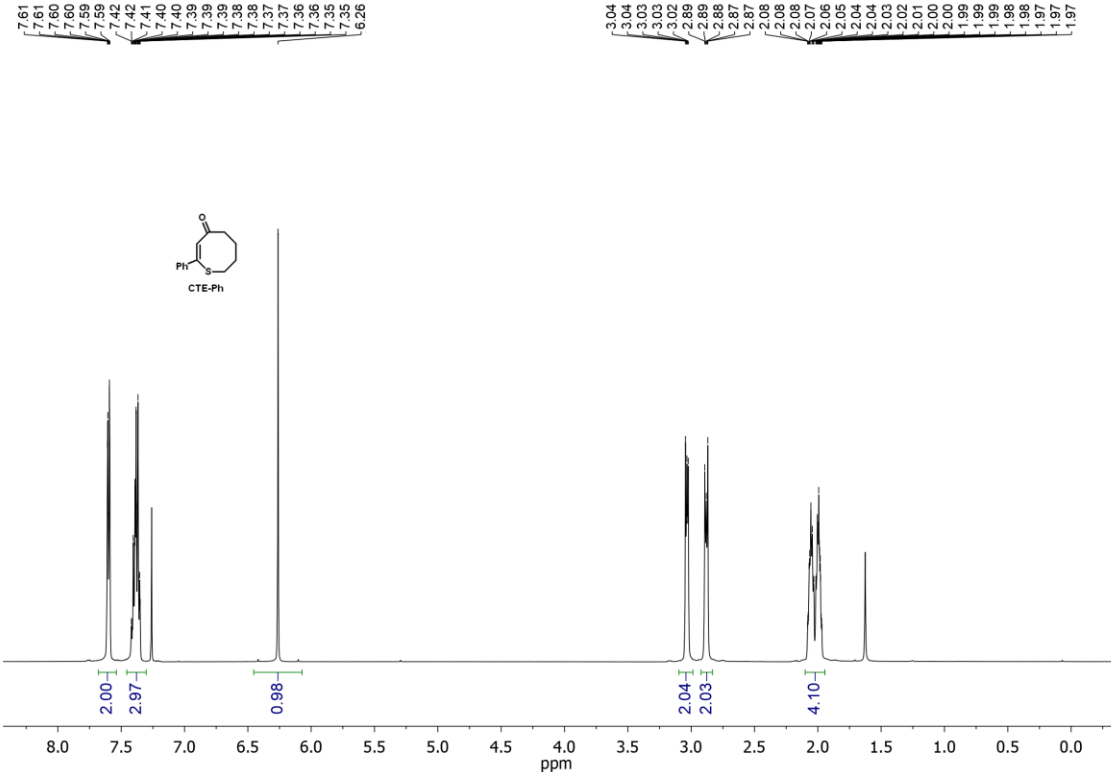

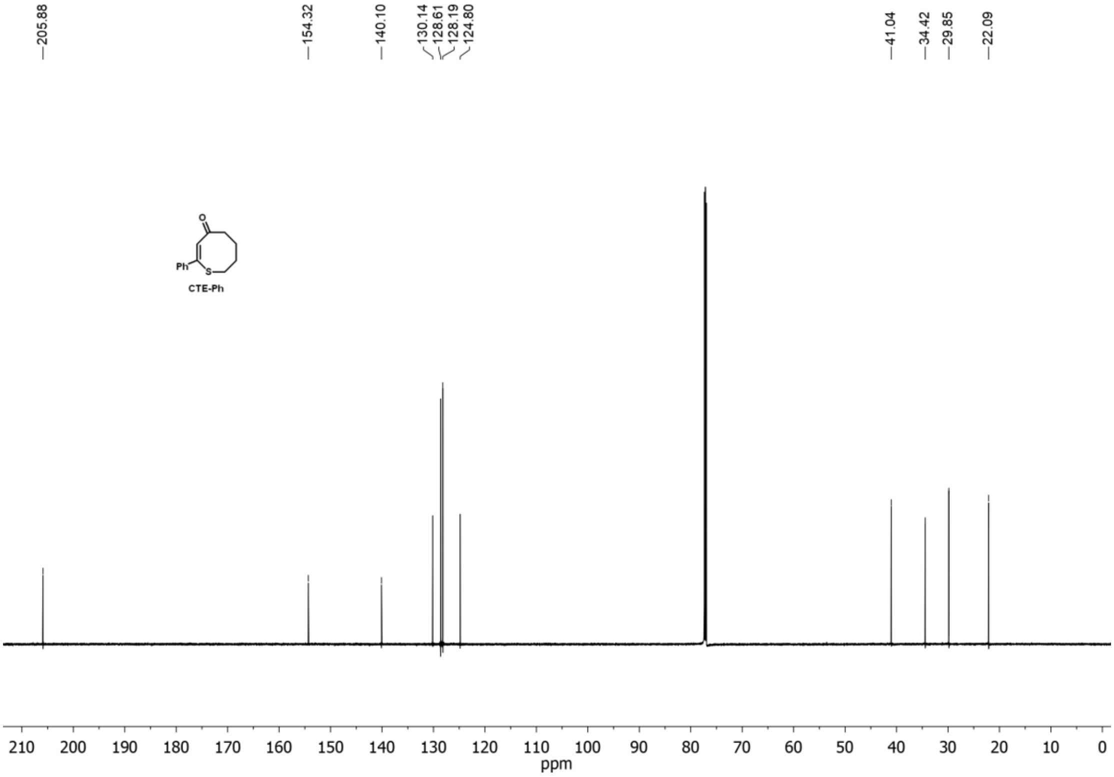

^{1}H NMR (500 MHz, $CDCl_3$) and ^{13}C NMR (176 MHz, $CDCl_3$) spectra for CTE-*n*-Bu

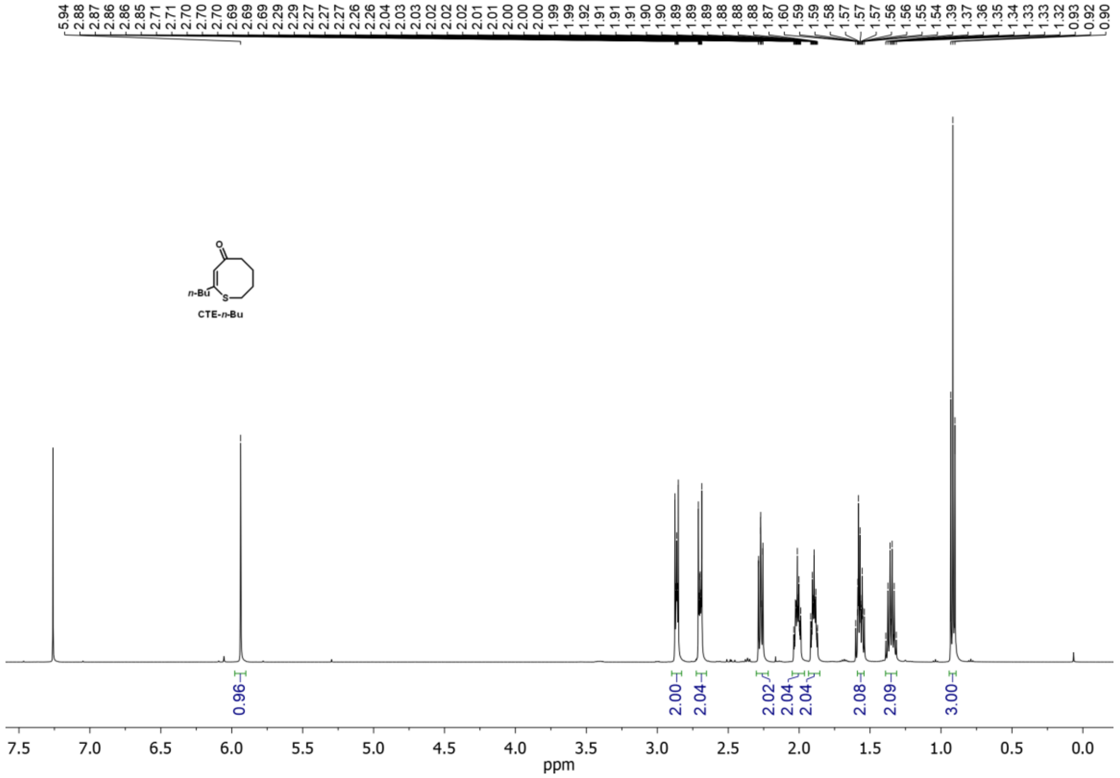

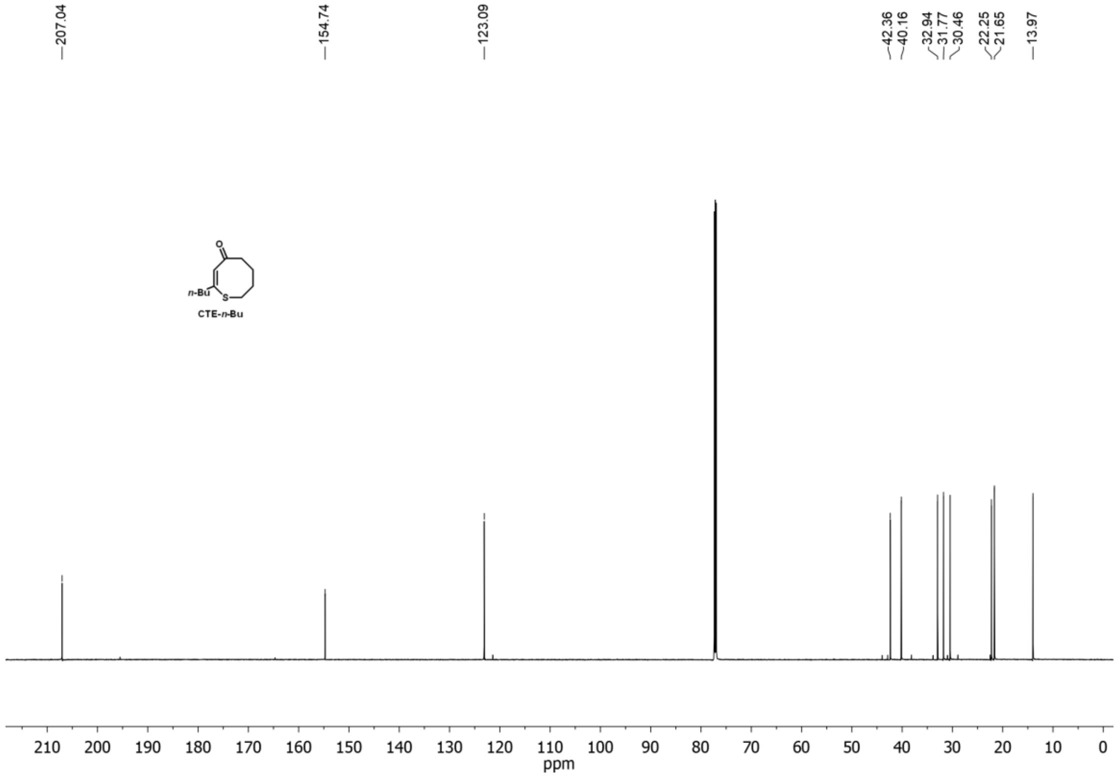

^{1}H NMR (500 MHz, CDCl$_3$) and ^{13}C NMR (176 MHz, CDCl$_3$) spectra for CTE-Fr

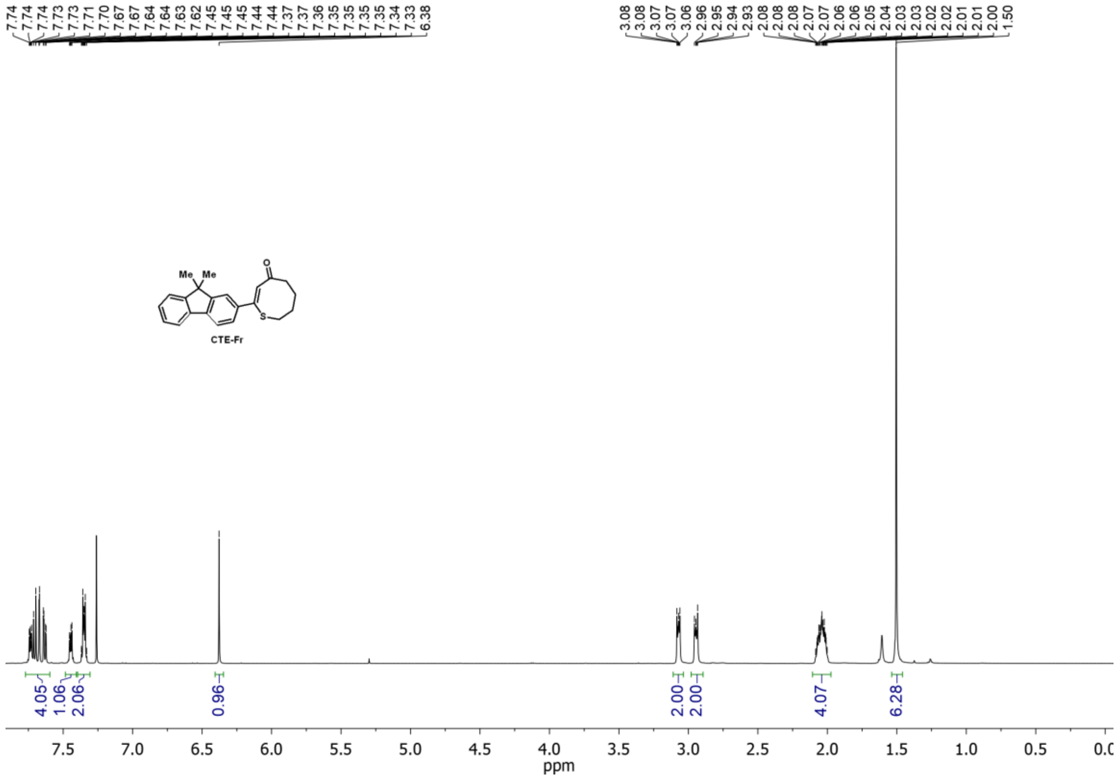

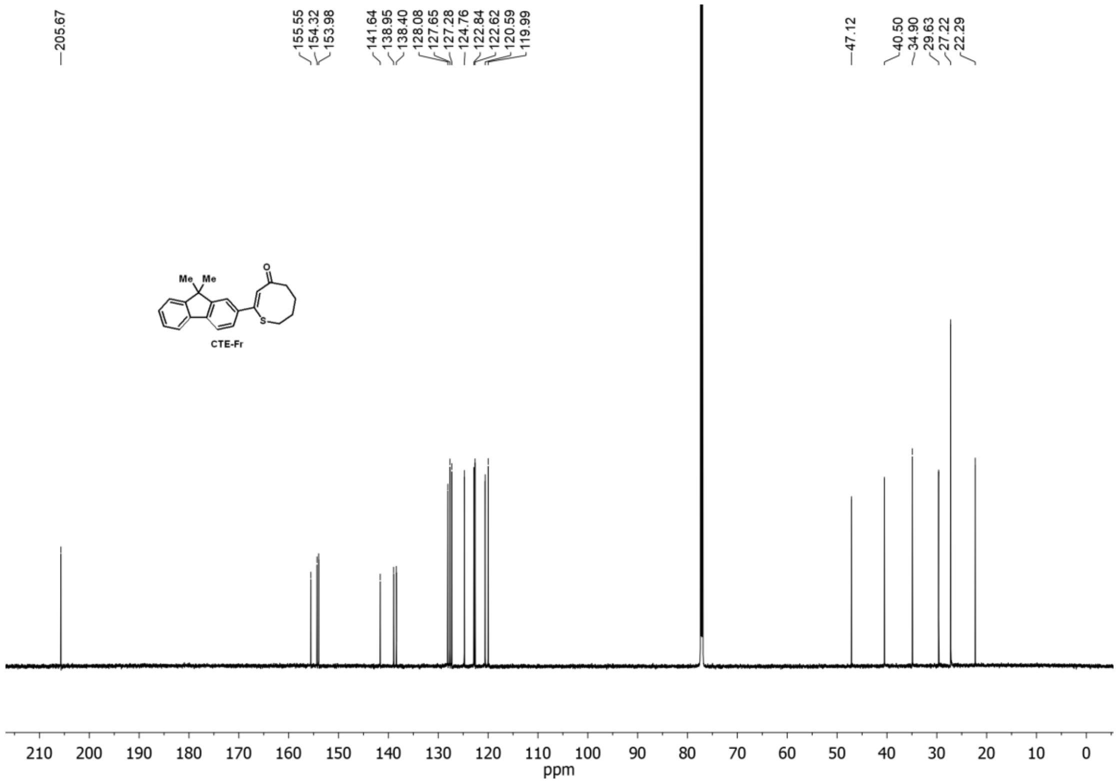

^1H NMR (400 MHz, $CDCl_3$) and ^{13}C NMR (176 MHz, $CDCl_3$) spectra for CTE-CO_2Et

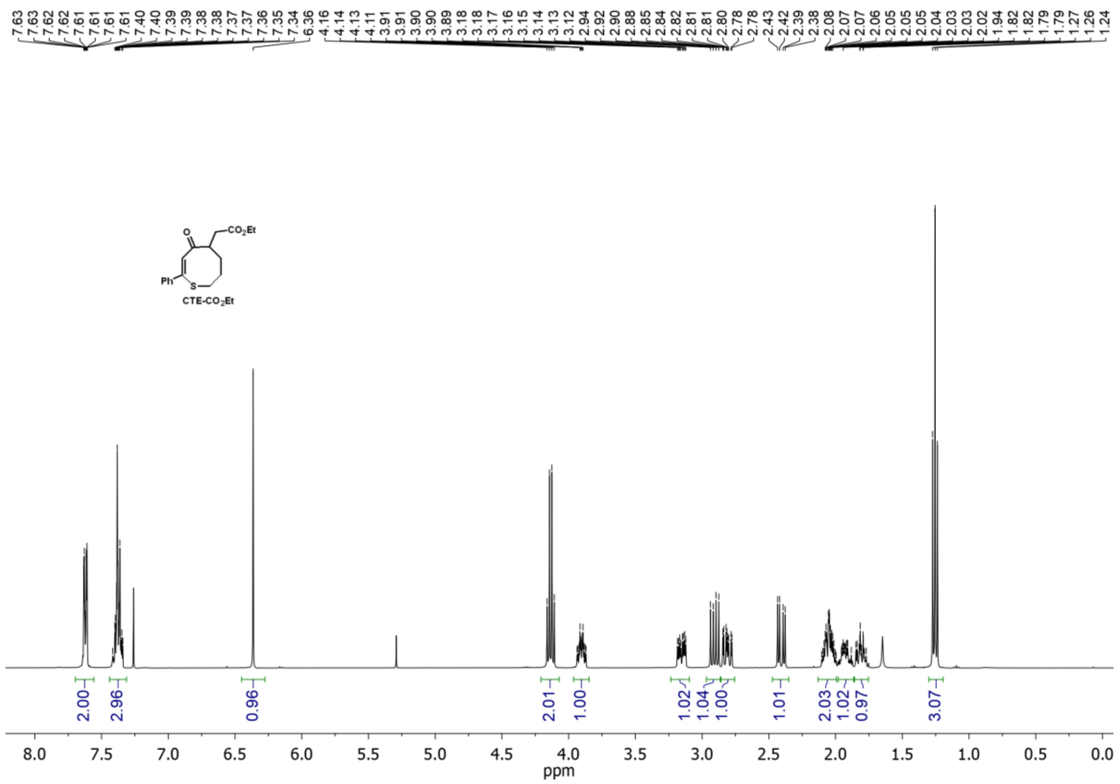

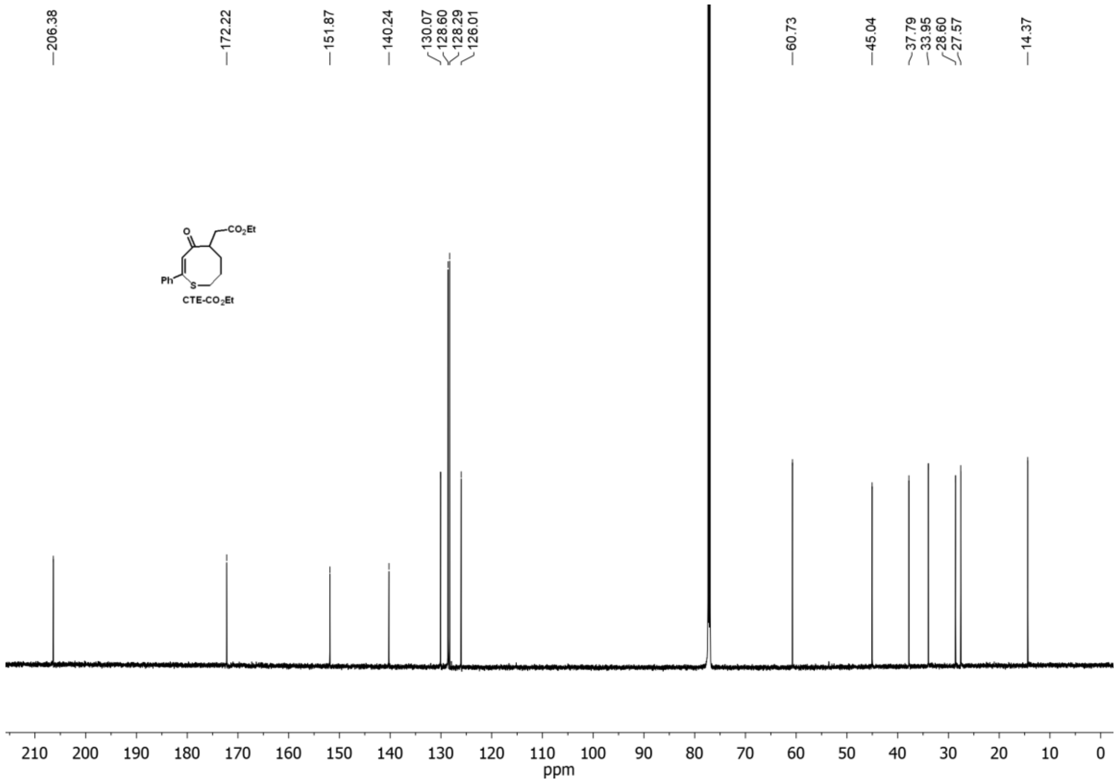

^{1}H NMR (700 MHz, $CDCl_3$) and ^{13}C NMR (176 MHz, $CDCl_3$) spectra for CTE-Cl

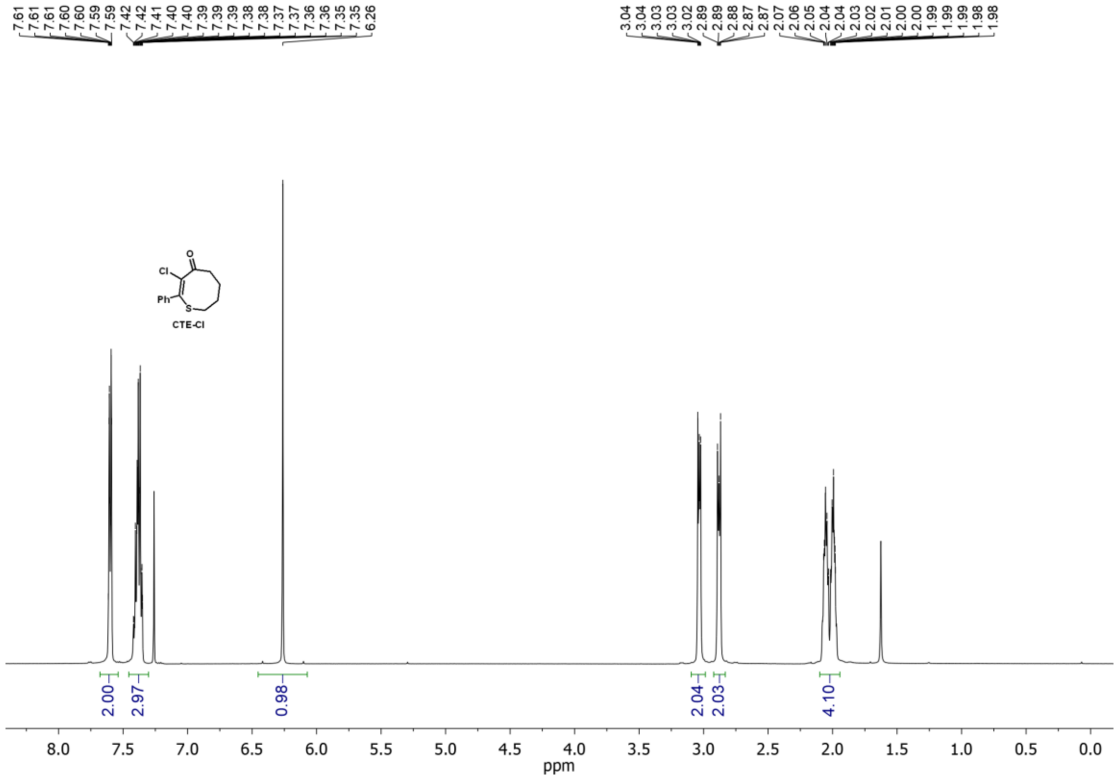

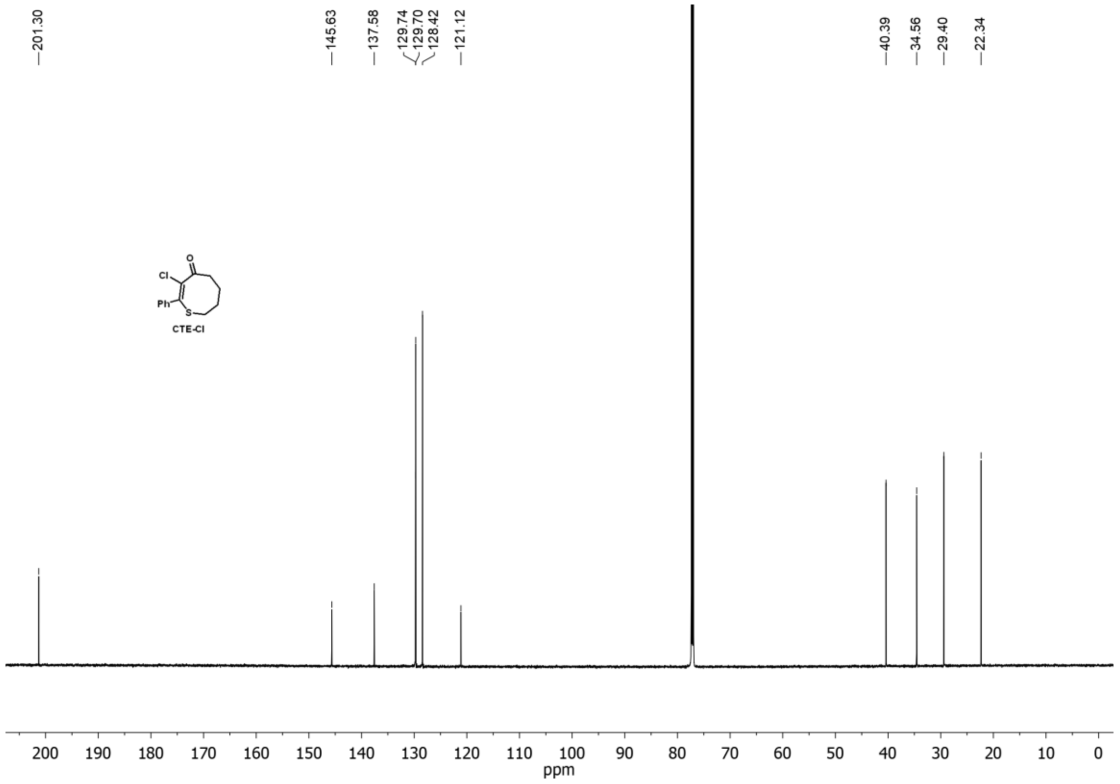

^{1}H NMR (700 MHz, $CDCl_3$) and ^{13}C NMR (176 MHz, $CDCl_3$) spectra for PCTE-Ph

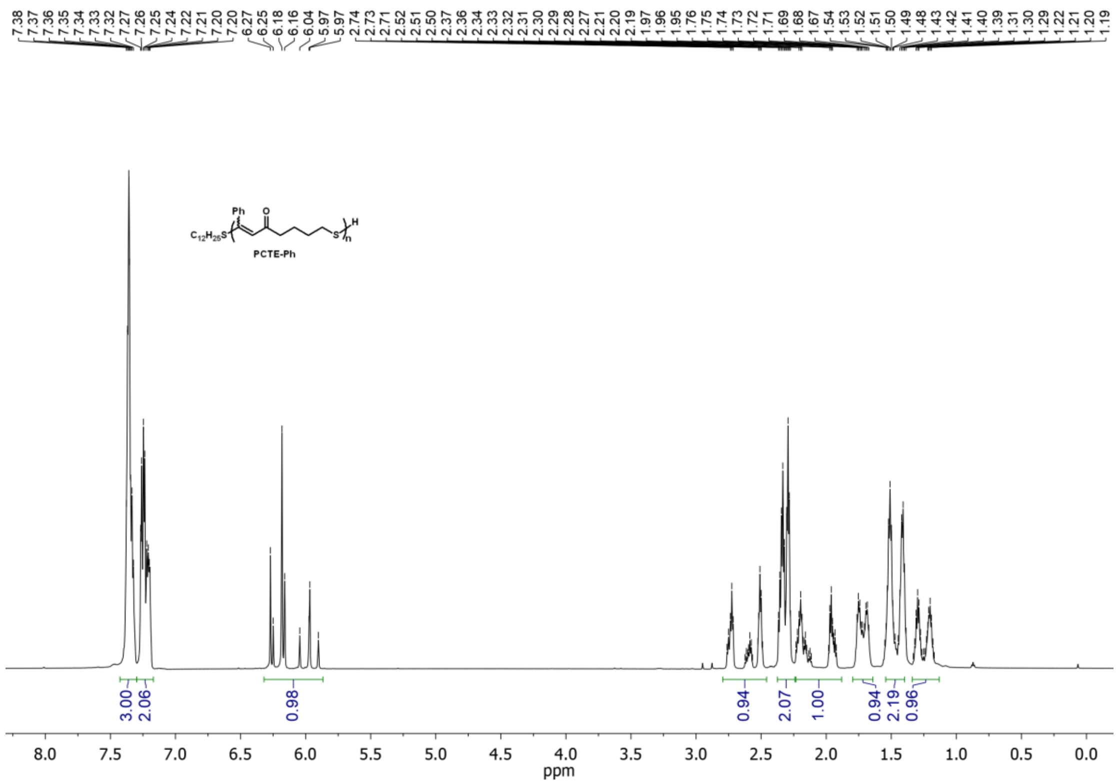

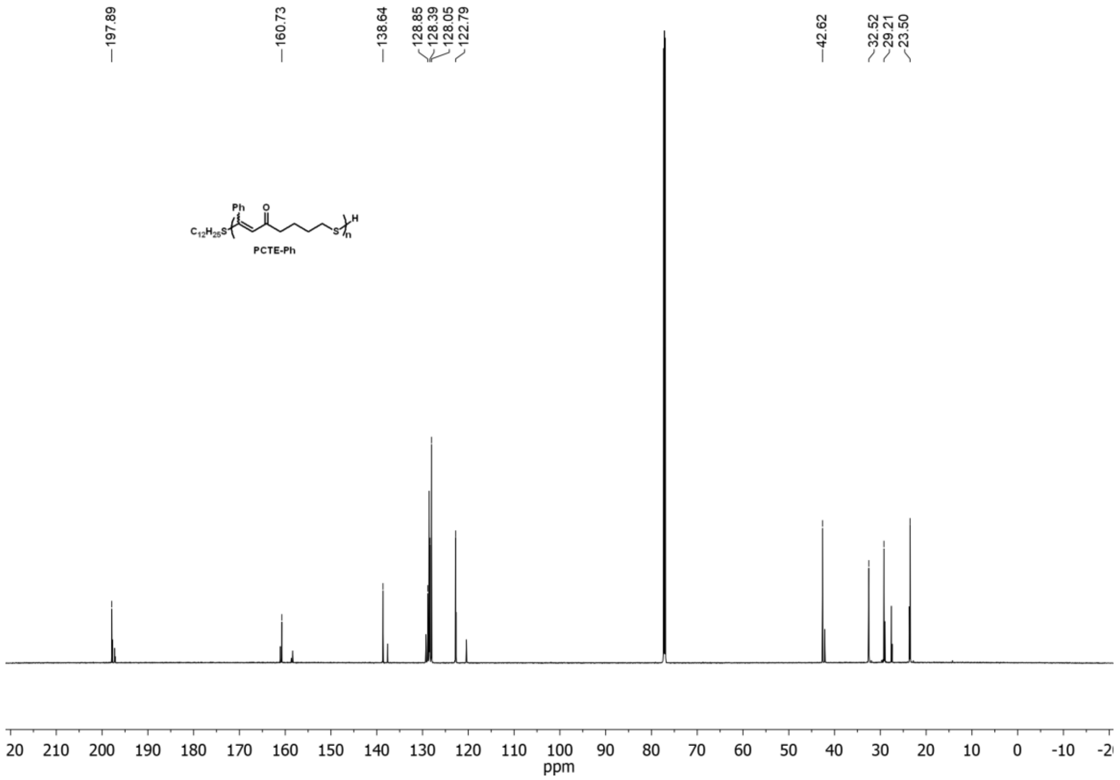

^{1}H NMR (700 MHz, $CDCl_3$) and ^{13}C NMR (176 MHz, $CDCl_3$) spectra for PCTE-*n*-Bu

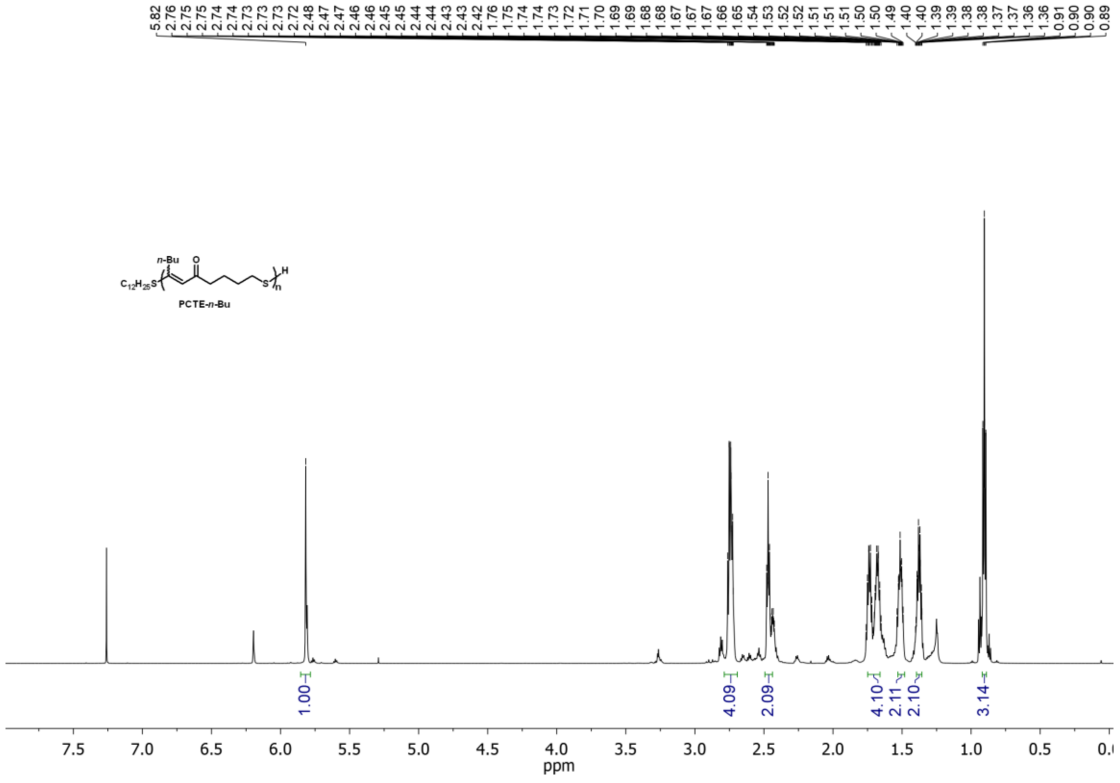

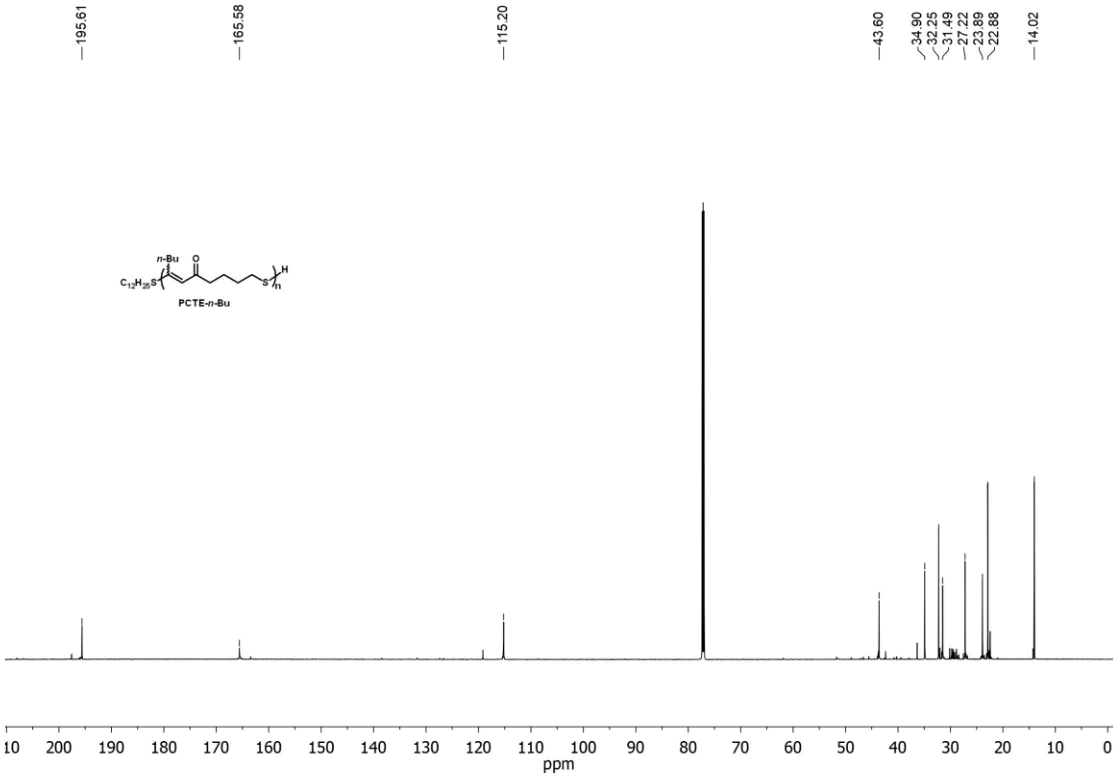

^{1}H NMR (700 MHz, CDCl$_3$) and ^{13}C NMR (176 MHz, CDCl$_3$) spectra for PCTE-CO$_2$Et

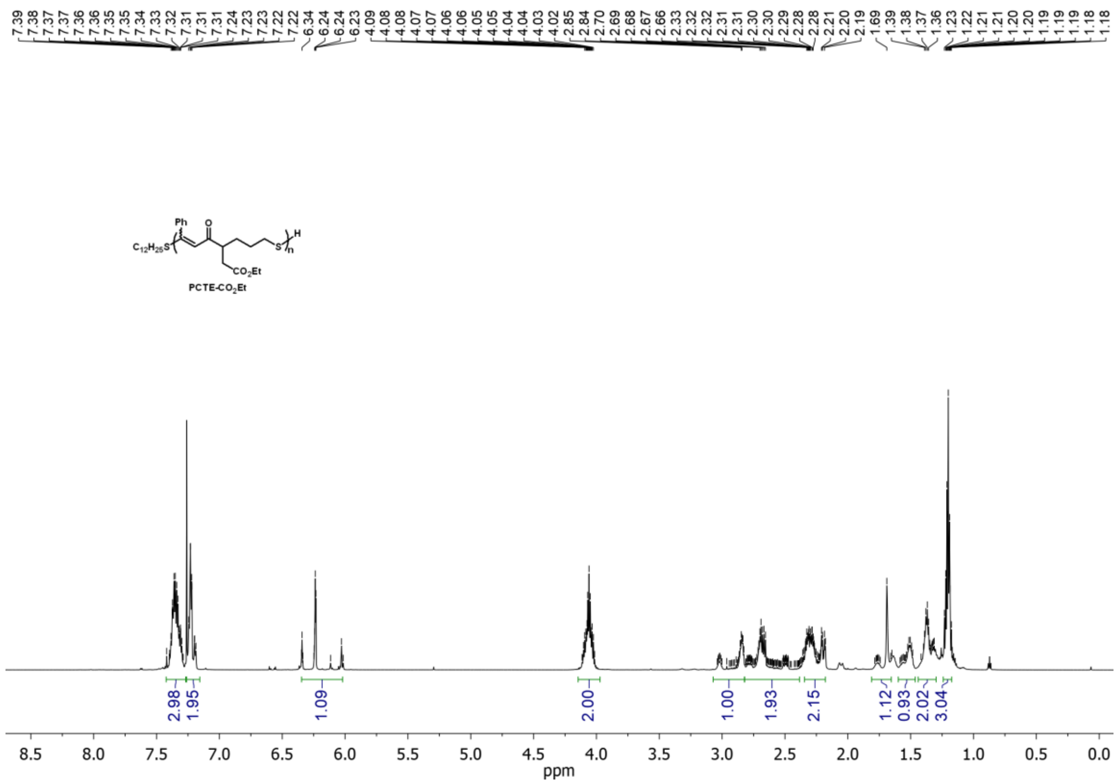

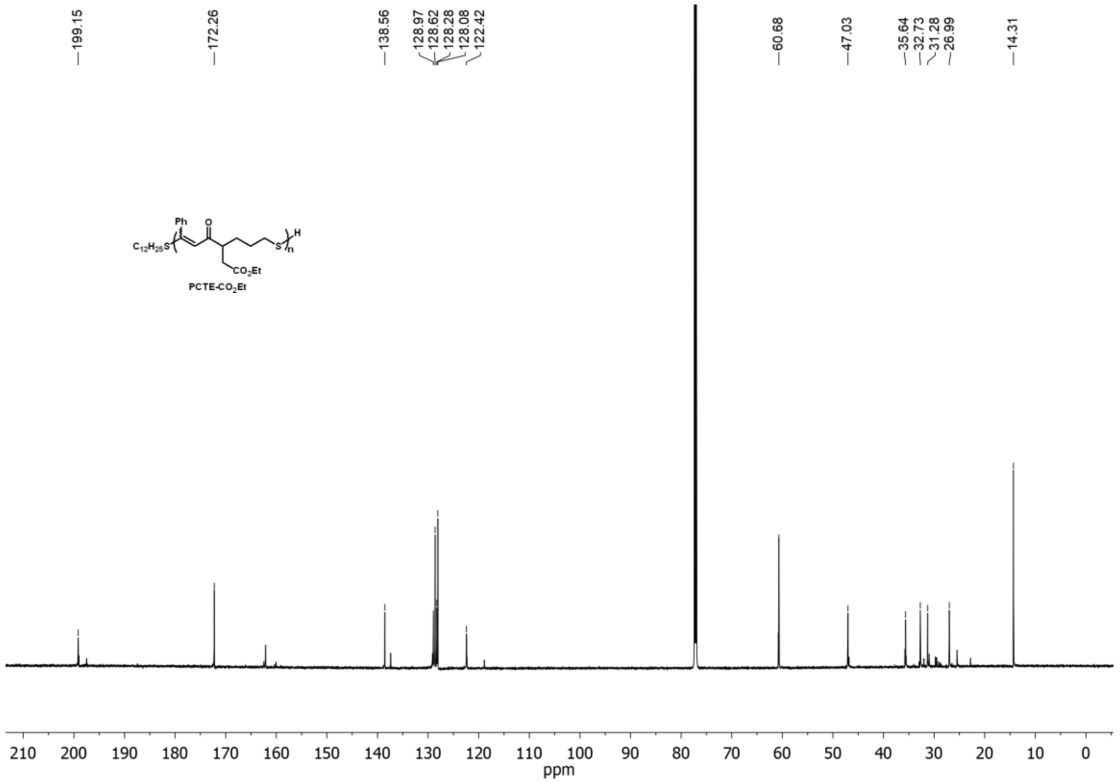

^{1}H NMR (700 MHz, $CDCl_3$) and ^{13}C NMR (176 MHz, $CDCl_3$) spectra for PCTE-Cl

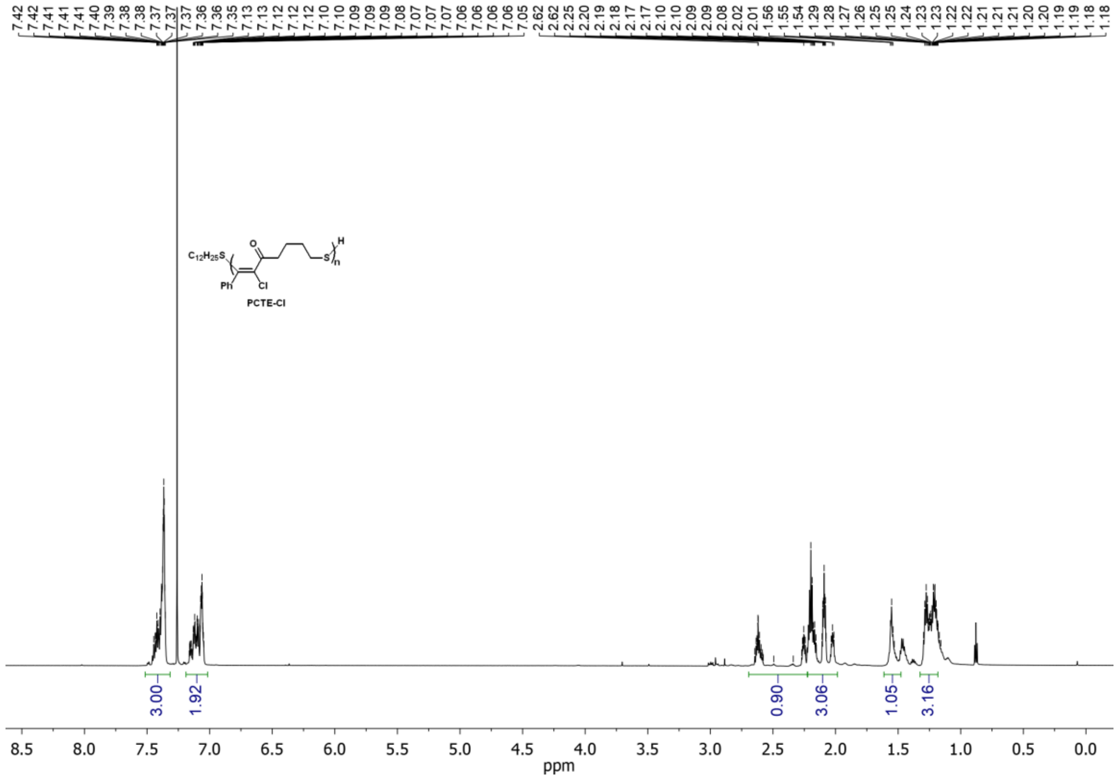

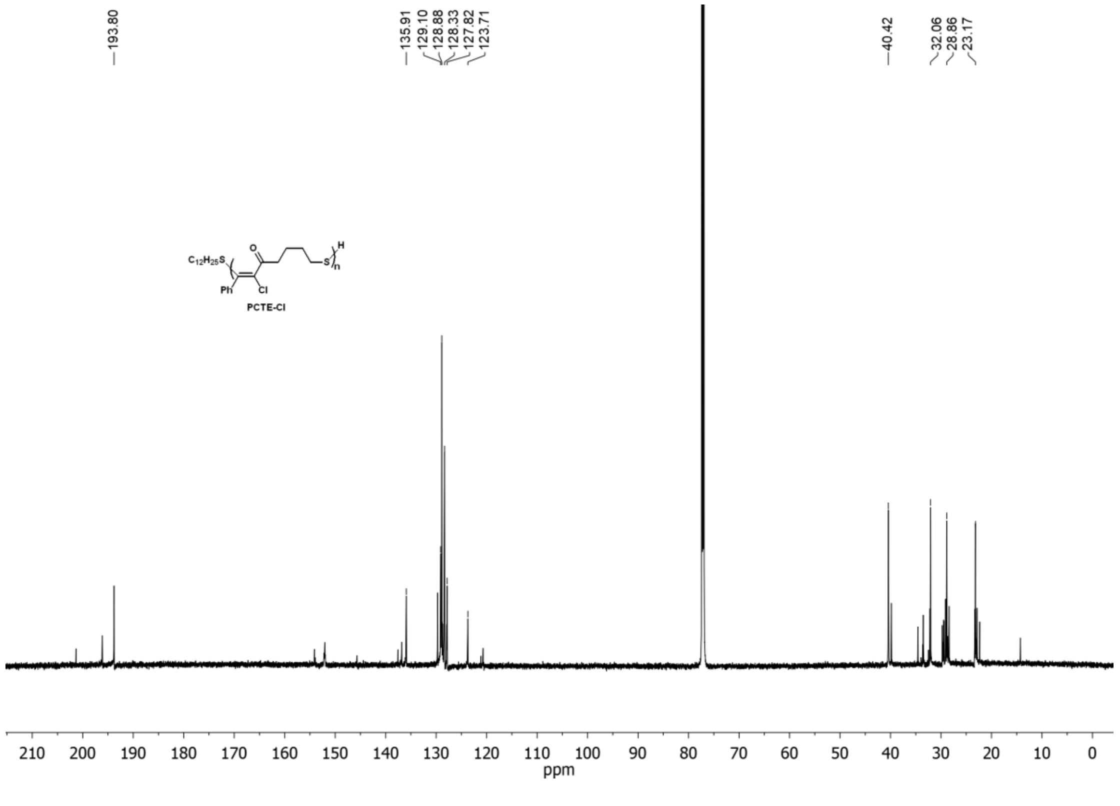

^{1}H NMR (700 MHz, $CDCl_3$) and ^{13}C NMR (176 MHz, $CDCl_3$) spectra for PCTE-Fr

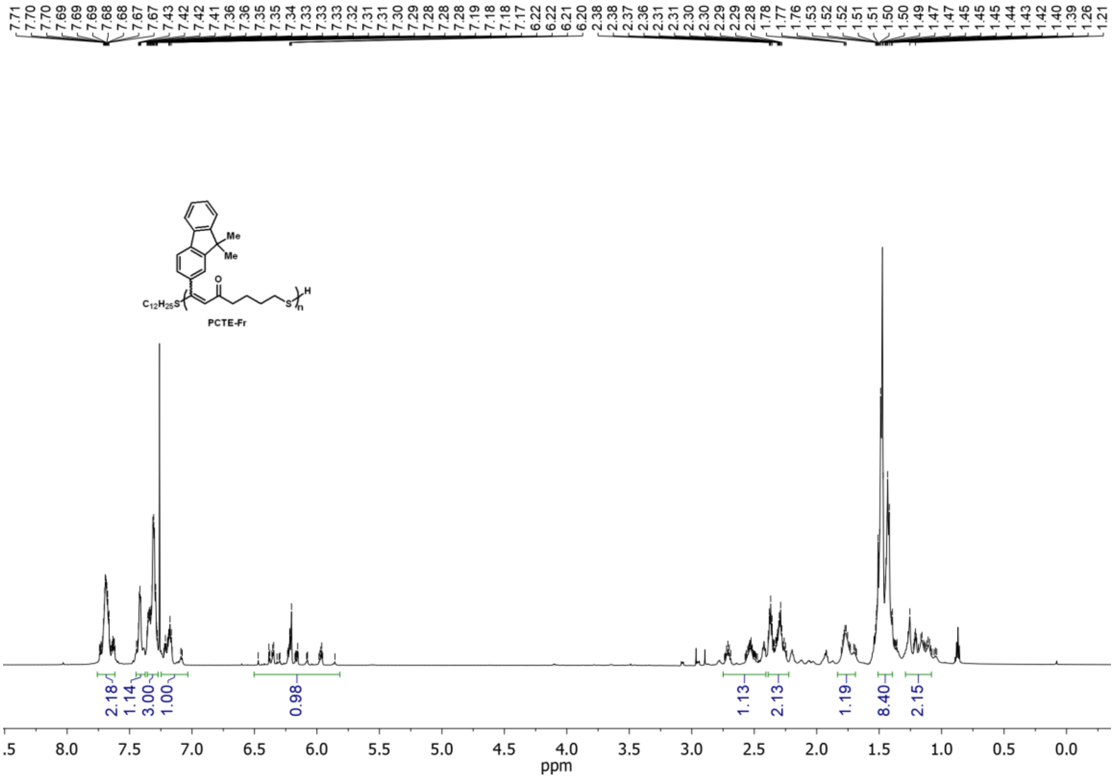

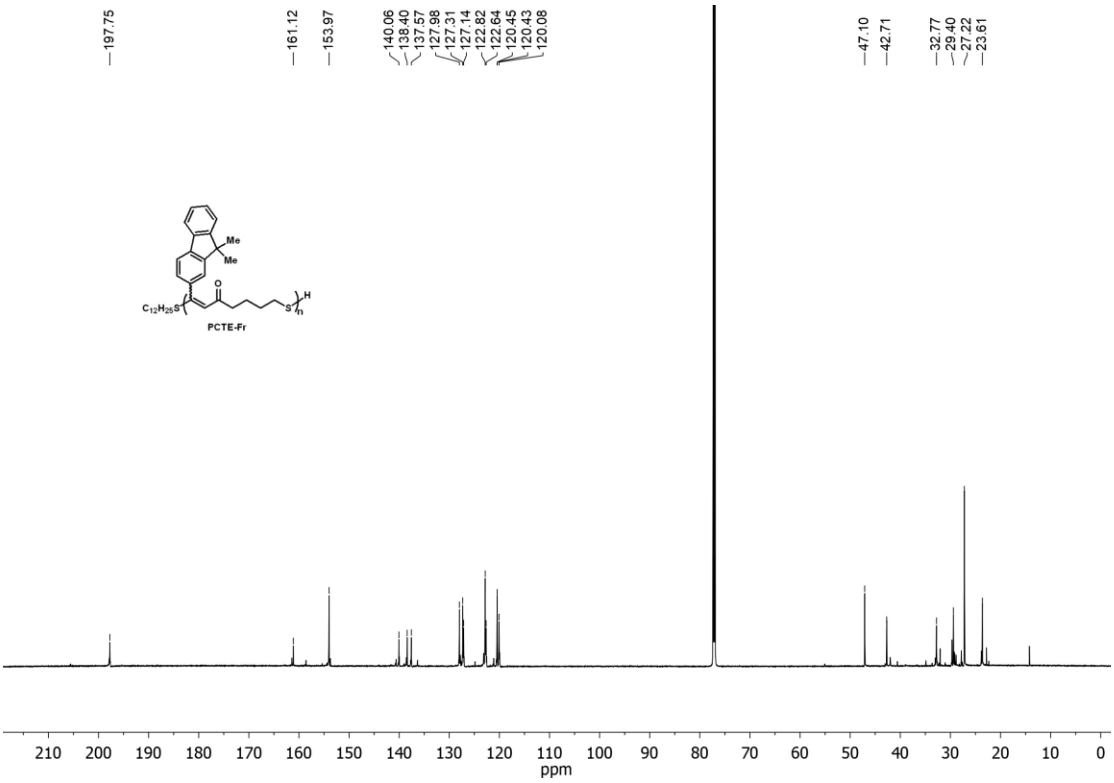

12. References

1. Oh, K.; Kim, H.; Cardelli, F.; Bwititi, T.; Martynow, A. M. *J. Org. Chem.* **2008**, *73*, 2432−2434.
2. Dolomanov, O.V.; Bourhis, L. J.; Gildea, R. J.; Howard, J. A. K.; Puschmann, H. *J. Appl. Cryst*. **2009**, *42*, 339−341.
3. Bourhis, L. J.; Dolomanov, O. V.; Gildea, R. J.; Howard, J. A. K.; Puschmann, H. *Acta Cryst*. **2015**, *A71*, 59−75.
4. Sheldrick, G.M. *Acta Cryst*. **2015**, *C71*, 3−8.
